# Regulation of Oncogene Expression in T-DNA-Transformed Host Plant Cells

**DOI:** 10.1371/journal.ppat.1004620

**Published:** 2015-01-23

**Authors:** Yi Zhang, Chil-Woo Lee, Nora Wehner, Fabian Imdahl, Veselova Svetlana, Christoph Weiste, Wolfgang Dröge-Laser, Rosalia Deeken

**Affiliations:** 1 Julius-von-Sachs-Institute, Department of Molecular Plant Physiology and Biophysics, University of Wuerzburg, Wuerzburg, Germany; 2 Leibniz Institute of Plant Biochemistry, Halle /Saale, Germany; 3 Julius-von-Sachs-Institute, Pharmaceutical Biology, University of Wuerzburg, Wuerzburg, Germany; 4 Institute of Biochemistry and Genetics, Ufa Scientific Centre of Russian Academy of Sciences, Ufa, Russia; University of Toronto, CANADA

## Abstract

Virulent *Agrobacterium tumefaciens* strains integrate their T-DNA into the plant genome where the encoded agrobacterial oncogenes are expressed and cause crown gall disease. Essential for crown gall development are *IaaH* (indole-3-acetamide hydrolase), *IaaM* (tryptophan monooxygenase) and *Ipt* (isopentenyl transferase), which encode enzymes for the biosynthesis of auxin (*IaaH*, *IaaM*) and cytokinin (Ipt). Although these oncogenes are well studied as the tumor-inducing principle, nothing is known about the regulation of oncogene expression in plant cells. Our studies show that the intergenic regions (IGRs) between the coding sequences (CDS) of the three oncogenes function as promoters in plant cells. These promoters possess a eukaryotic sequence organization and *cis*-regulatory elements for the binding of plant transcription factors. WRKY18, WRKY40, WRKY60 and ARF5 were identified as activators of the *Ipt* promoter whereas *IaaH* and *IaaM* is constitutively expressed and no transcription factor further activates their promoters. Consistent with these results, the *wrky* triple mutant plants in particular, develops smaller crown galls than wild-type and exhibits a reduced *Ipt* transcription, despite the presence of an intact *ARF5* gene. *WRKY40* and *WRKY60* gene expression is induced by *A. tumefaciens* within a few hours whereas the *ARF5* gene is transcribed later during crown gall development. The WRKY proteins interact with ARF5 in the plant nucleus, but only WRKY40 together with ARF5 synergistically boosts the activation of the *Ipt* promoter in an auxin-dependent manner. From our data, we propose that *A. tumefaciens* initially induces *WRKY40* gene expression as a pathogen defense response of the host cell. The WRKY protein is recruited to induce *Ipt* expression, which initiates cytokinin-dependent host cell division. With increasing auxin levels triggered by ubiquitous expression of *IaaH* and *IaaM*, ARF5 is activated and interacts with WRKY40 to potentiate *Ipt* expression and balance cytokinin and auxin levels for further cell proliferation.

## Author Summary

Crown gall development requires the expression of agrobacterial genes in the plant host. These genes are transferred by the T-DNA of the plant pathogen *Agrobacterium tumefaciens* and include the oncogenes *IaaH, IaaM* and *Ipt*, which, according to the tumor-inducing principle, are essential for crown gall development. The oncogenes are involved in auxin and cytokinin production. This results, when at appropriate hormone ratios, in enhanced cell proliferation. The T-DNA transformation process and the encoded oncogene enzymes have been intensively studied, but knowledge of oncogene expression in plant cells and the regulatory host factors is missing. We set out to fill this gap, providing evidence that expression of the *Ipt* gene is host-cell controlled, whereas the *IaaH* and *IaaM* genes are ubiquitously expressed at low levels in T-DNA transformed tissue. This is achieved by *A. tumefaciens*, which first hijacks transcription factors of the plant pathogen response pathway to activate *Ipt* oncogene expression and initiates cell proliferation. With increasing auxin levels during the infection process, a transcription factor of the auxin-signaling pathway is recruited, potentiating *Ipt* gene expression. Thus, for crown gall development, two host-signaling pathways are combined through the interaction of transcription factors that adjust the ratio of cytokinin to auxin.

## Introduction

*Agrobacterium tumefaciens* is a pathogenic bacterium that infects several plant species. A region in the tumor inducing (Ti) plasmid, the transfer DNA (T-DNA), is integrated into the plant genome causing crown gall disease [1]. There are essentially two groups of genes encoded on the T-DNA of virulent *A. tumefaciens* strains [2]. The first is responsible for producing opines, so providing a carbon and nitrogen source for *A. tumefaciens*, with the second group expressing the oncogenes required for crown gall development. These oncogenes include *IaaH, IaaM, Ipt, gene 6b* and *gene 5*. It is assumed that although *gene 6b* and *gene 5* are expendable, *IaaH, IaaM* and *Ipt* are crucial for crown gall development [3–5]. *IaaH* and *IaaM* code for enzymes that catalyze biosynthesis of auxin and *Ipt* mediates cytokinin biosynthesis [5,6]. *IaaM* encodes a tryptophan monooxygenase that converts tryptophan (Trp) into indole-3-acetamide (IAM), and *IaaH* an indole-3-acetamide hydrolase, converts IAM into indole-3-acetic acid (IAA) [7–9]. Ipt (isopentenyl transferase) catalyzes the rate-limiting step in cytokinin biosynthesis [2,5,10]. Cytokinins can also be synthesized in *A. tumefaciens* cells by the chromosomal encoded *miaA* enzyme [11,12] and the trans-zeatin synthesizing (tzs) enzyme encoded on the nopaline-type pTi-plasmid [13–15]. *A. tumefaciens* secretes auxin and cytokinin from the cells to initiate crown gall development [16] and pretreatment of plant tissues with auxin and cytokinin promotes *A. tumefaciens*-mediated transformation efficiency [14,17,18]. Very recently it was shown that cytokinins secreted by *A. tumefaciens* repress a Myb transcription factor in host plant cells, resulting in an enhanced transformation efficiency [18].

The increased production of auxin and cytokinin in T-DNA transformed plant cells expressing the *IaaH, IaaM* and *Ipt* oncogenes induces cell proliferation and differentiation [19,20]. Therefore, a T-DNA harboring plant cell needs to initiate transcription of the three oncogenes in order to express their function. In eukaryotic cells, the RNA polymerase II complex mediates transcription of mRNAs from protein-coding genes. This complex recognizes the TATA box and the transcription start site (TSS) [21] within upstream promoter regions that drive the expression of the downstream coding sequence (CDS). These two sequence features build the core promoter and this is sufficient to transcribe a gene [21]. TATA boxes were predicted to be present 5’ upstream of the CDS of the *IaaH, IaaM* and *Ipt* oncogenes [22–24]. In addition to initiation of transcription by the RNA polymerase II complex, expression of eukaryotic genes is usually regulated by transcription factors. These bind to regulatory sequence elements localized in the promoter regions of many eukaryotic genes and are oriented in a sense or anti-sense direction distant from the TSS [21]. For the *Ipt* gene of the octopine Ti plasmid pTiAch5, a 184 bp fragment upstream of the CDS is sufficient for transcription in plant cells [25]. In particular, the region between −185 and −139 bp from the translational start codon are essential [26]. Within that region, the 30 bp sequence *cyt-1* binds an as yet unknown protein from tobacco nuclear protein extracts, designated CBF (*cyt-1* binding factor) [27]. This suggests that expression of the agrobacterial oncogenes can be regulated by host transcription factors that await discovery.

A well-known response of plants to microbial pathogens is the microbe associated molecular pattern (MAMP)-induced innate immunity response, which includes expression of several WRKY transcription factors [28]. The expression profiles of 72 WRKY genes in *Arabidopsis* revealed that 49 genes are responsive to salicylic acid (SA) and pathogen treatment [29]. The WRKY transcription factor binding elements, the W-boxes (TGAC), are present in many defense related gene promoters [28]. In addition to the induction of pathogen defense responses, crown gall development requires cell proliferation and differentiation, such as vascularization [30]. These developmental programs are synergistically controlled by auxin and cytokinin signaling pathways that lead to changes in the regulation of gene expression. The expression of some auxin responsive factor (ARF) genes is induced by auxin, particularly in developing embryos and vascular tissues [31]. ARFs are known to induce the transcription of genes in an auxin-dependent manner by binding to auxin response elements (AuxREs) in auxin responsive promoters [31,32]. The regulation of ARF function involves auxin/indole acetic acid (Aux/IAA) proteins and TIR1 (transport inhibitor response 1) [33,34]. Aux/IAA proteins interact and repress the transcriptional activity of ARFs [35,36]. The F-box auxin receptor TIR1 is part of the SCF^TIR^ ubiquitin ligase complex [37,38]. At increasing auxin concentrations, Aux/IAA proteins are recognized and ubiquitinylated by the SCF^TIR^ complex and subsequently degraded by the 26S proteasome [39,40]. The de-repressed ARF proteins can activate target promoters.

This study focuses on the transcriptional regulation of the *A. tumefaciens* genes *IaaH, IaaM* and *Ipt* in the host plant. The intergenic regions between the CDSs of *IaaH, IaaM* and *Ipt* of the virulent T-DNA of *A. tumefaciens* strain C58 (pTiC58, AE007871) showed promoter activity in *Arabidopsis* cells. The *IaaH* and *IaaM* genes involved in auxin biosynthesis in T-DNA transformed cells, were ubiquitously expressed at low levels. In contrast, the *Ipt* promoter was activated by the transcription factor WRKY40 (AT1G80840), a transcription factor that responded rapidly to *A. tumefaciens* infection. WRKY40 together with ARF5 (AT1G19850), which is part of an auxin-dependent signaling pathway, boosted *Ipt* promoter activity in an auxin dependent manner. This enhanced activity correlated with *cis*-regulatory elements such as W-boxes and AuxREs in the *Ipt* promoter and the protein interaction of WRKY40 with ARF5. Our findings suggest that *A. tumefaciens* recruits the WRKY-dependent pathogen defense pathway to activate *Ipt* gene expression. This can be substantially increased when the auxin-dependent developmental process mediated by ARF5 is switched on.

## Results

### The intergenic regions between the oncogenes function as promoters in plant cells

**Figure 1:**
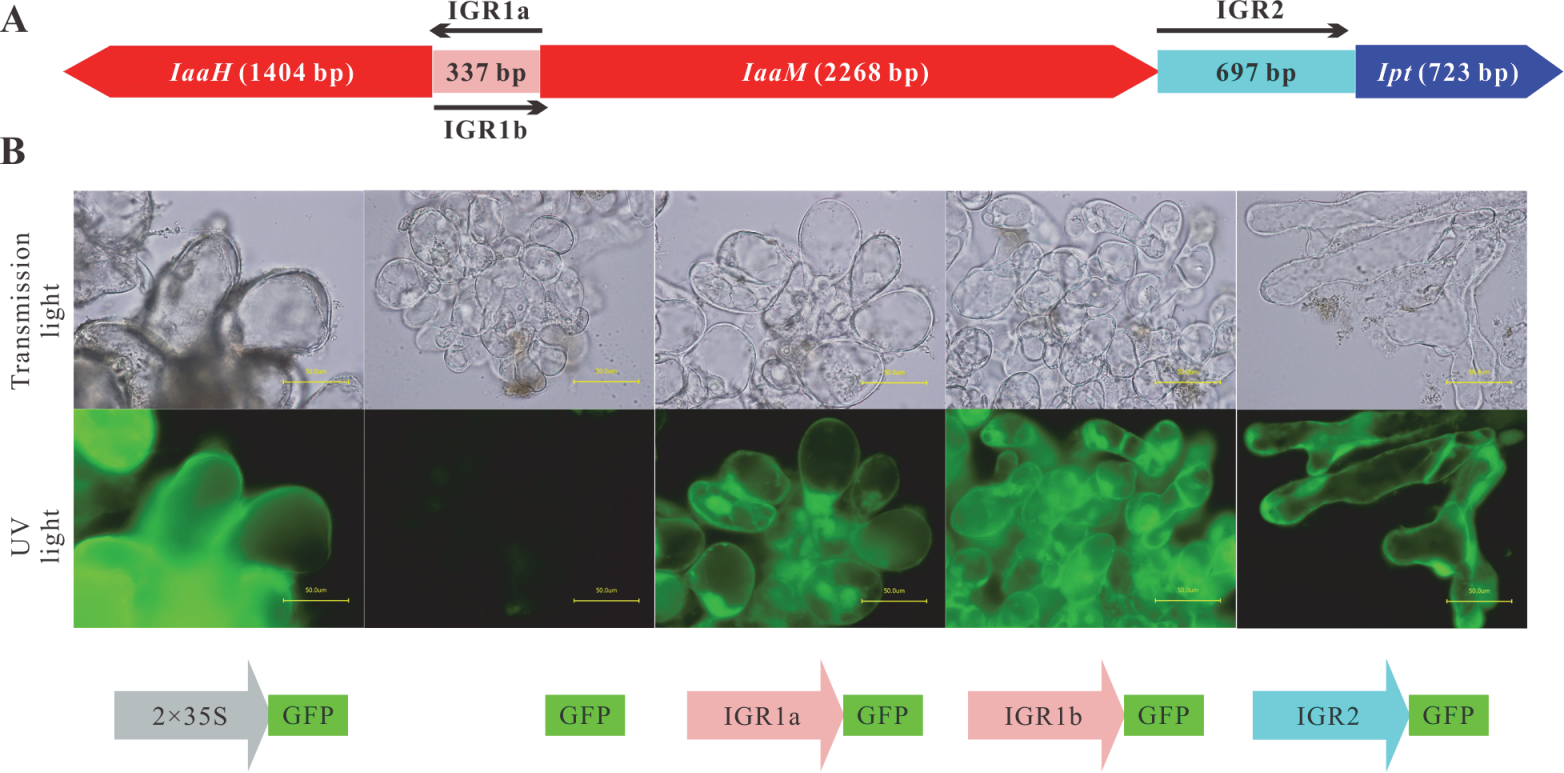
IGR1 and IGR2 function as promoters in *Arabidopsis* cells. (A) Arrangement of the coding sequences of the *IaaH, IaaM* and *Ipt* oncogenes and the intergenic regions (IGRs) in the T-DNA region of the Ti plasmid of *A. tumefaciens* strain C58, pTiC58. (B) *Arabidopsis* crown gall callus cells expressing the green fluorescing protein (GFP) under the control of IGR1a (*IGR1a::GFP*), IGR1b (*IGR1b::GFP*) and IGR2 (*IGR2::GFP*). IGR1 was used in two orientations; one is upstream of *IaaH* CDS (IGR1a) and the other upstream of *IaaM* (IGR1b). The universal cauliflower mosaic virus promoter was used as a positive control (*2× CaMV35S::GFP*) and the *GFP* CDS without promoter, as the negative control (*GFP*). Images show crown gall callus cells in the transmission microscopy (top row) and the UV light mode (bottom row, excitation: 490 nm, emission: 510 nm). The UV-light intensity used for excitation is the same for both pictures. Bars, 50 μm.

To discover how the expression of the agrobacterial oncogenes *IaaH, IaaM* and *Ipt* is regulated in plant cells, we analyzed the structure of the T-DNA region of the nopaline-type Ti plasmid pTiC58. The CDS of the three oncogenes are sequentially arranged and interrupted by two non-coding intergenic regions (IGR1 and IGR2; Fig. 1A). The *IaaM* and *Ipt* genes are transcribed from the sense strand and the *IaaH* gene is encoded on the opposite strand. If IGR1 functions as a promoter for both the *IaaH* and *IaaM* oncogenes, it must be a bidirectional promoter: one direction being 5’ upstream of the TSS of the *IaaH* CDS (IGR1a) and the other, 5’ upstream of *IaaM* (IGR1b).

To prove whether the IGRs function as promoters in plant cells, the complete IGR sequences were fused with the CDS of the green fluorescent protein (GFP) in a binary vector. The IGR1a and IGR1b sequences included the 5’ untranslated regions (5’ UTR) of both the *IaaH* and *IaaM* genes, whereas IRG2 contained the 3’ UTR of *IaaM* and 5’ UTR of the *Ipt* gene. We generated stable transformed *Arabidopsis* crown gall tumor cell lines by infecting *Arabidopsis* root segments with the virulent *A. tumefaciens* strain C58, which, in addition to their pTiC58, harbor a binary vector with the *IGR::GFP* constructs. Detection of GFP fluorescence in the *IGR1a::GFP, IGR1b::GFP* and *IGR2::GFP* crown gall cell lines demonstrated that the IGRs drive GFP expression, so function as promoters in plant cells (Fig. 1B). Furthermore, as the IGR1 sequence is a bidirectional promoter, it can drive transcription of both the *IaaH* and *IaaM* genes.

Since IGR1a, IGR1b and IGR2 all function as promoters in eukaryotic cells, their sequences should contain the core promoter elements, such as the initiator (Inr) sequence and TATA box. To localize these in the promoters, we determined the TSSs of the *IaaH, IaaM* and *Ipt* genes using the 5’ rapid amplification of cDNA ends (5’ RACE) assay, finding that the translational start codon of the *IaaH, IaaM* and *Ipt* CDSs are at positions +12 bp, +26 bp and +44 bp in respect to the TSS (Table 1). Upstream of the TSSs, the typical eukaryotic Inr box (YYANWYY, TSS is underlined, Y = C/T, W = A/T, N = A/G/C/T) was present in the three promoter sequences. This is in agreement with the plant specific “YR Rule” (YR, TSS is underlined, Y = C/T, R = A/G [41,42]). The TATA boxes, the binding sites for the general transcription factor complex, are found in the promoter regions −25 bp to −35 bp and another feature of many eukaryotic promoters, the CAAT boxes, are localized approximately −70 bp upstream of the TSSs within the oncogene promoter regions (Table 1).

**Table 1:** *Cis*-regulatory sequence elements within the promoter of the oncogenes *IaaH, IaaM* and *Ipt* encoded on the T-DNA of the Ti plasmid from *A. tumefaciens* strain C58, pTiC58.

**Promoter (sequence)**	***IaaH*(−1 to −301)**	***IaaM*(−1 to −301)**	***Ipt fwd*(−1 to −654)**	***Ipt*rev (−1 to −654)**
**Positions of core promoter sequence elements**				
**Inr box** (YY**A**NWYY)	−2 CC**A**AACC +5	−2 CT**A**CACA +5	−2 CT**A**ATCC +5	
Start coden (ATG)	+12 ATG	+26 ATG	+44 ATG	
**TATA box**(TATAAA)	−36 TATATT −31 ^1^	−32 TAAATA −27 ^2^	−29 TATAAC −24 ^34, 56^	
**CAAT box**(GGNCAATCN)	−66 CCAAT −62 ^1^	−75 CCATT −71 ^2^	−72 GGTAAAGCC −64 ^3^ −49 AAGGAATCT −41 ^4^ ^, ^ ^5^	

**Number of transcription factor binding elements**				
MYBCORE (CNGTTR or AACGG)	0	2	5	3
MYB2AT (YAACKG)	0	0	1	2
DOFCOREZM (AAAG)	2	0	5	5
W-Box (TGAC)	1	0	4	2
EBOXBNNAPA (CANNTG)	0	0	4	4
ARR1 (GATT)	3	5	3	4
ARF (TGTCNC or TGTCTN)	0	1	2	3

Positive numbers indicate the positions downstream and negative numbers the positions upstream of the TSSs (+1). Y = C/T, K = G/T, W = A/T, R = A/G, N = A/G/C/T.

1. [23]

2. [24]

3. [22]

4. [72]

5. [73]

6. [74]

To ascertain whether the regulatory promoter elements of pTiC58 are conserved, we performed a sequence alignment with the promoter and 5’ untranslated regions (5’ UTRs) of the three oncogenes from different Ti plasmids. We compared the upstream sequences of the three oncogene CDSs of the Ti plasmids from two nopaline-types (pTiC58, pTiSAKURA), three octopine-types (pTiA6NC, pTiAch5, pTi15955) and one agropine-type (pTiBo542). The alignment shows that the TSSs (arrows), TATA boxes and CAAT boxes of the promoters for *IaaH* (S1 Fig.), *IaaM* (S2 Fig.) and *Ipt* (S3 Fig.) are conserved between the pTi plasmids of the different *A. tumefaciens* strains. In contrast, two TATA boxes are present 5’ upstream of the CDS in the *Ipt* genes from the octopine Ti plasmids (S3 Fig.). In the *Ipt* promoter of pTiC58, two CAAT boxes were predicted (S3 Fig.), one of which (GGTAAAGCC, from −72 to −64 bp) is conserved and also found in other nopaline type and in the octopine type pTi plasmids, but not in the agropine type *Ipt* promoter where no CAAT box was predicted. The second CAAT box (AAGGAATCT, −49 to −41 bp) is specific for the *Ipt* promoters of the nopaline type Ti-plasmids (S3 Fig.). *Cis*-regulatory binding elements for transcription factors were also determined in the *IaaH, IaaM* and *Ipt* promoters on the Watson and Crick strand using PLACE (http://www.dna.affrc.go.jp/PLACE/index.html) [43–45]. Several binding elements for different transcription factor families including MYB, DOF, WRKY, bHLH, ARR1 and ARF, were localized within the *Ipt* promoter (Table 1). In the *IaaH* and *IaaM* promoters, the binding element for the ARR1 (AT3G16857) transcription factor was dominant and there were eight ARR1 elements altogether.

To identify potential transcription factors that may be involved in enhancing the expression of the oncogenes, we analyzed existing microarray data of *Arabidopsis* crown galls [20,46], based on the *Arabidopsis* transcription factors listed in the Plant Transcription Factor Database v3.0 [47] (http://planttfdb.cbi.pku.edu.cn/index.php?sp=Ath). A total of 151 transcription factor genes were found to be differentially transcribed in inflorescence stems inoculated with the virulent *A. tumefaciens* strain C58 compared to non-inoculated stems (S1 Table; fold change ≥ 2 or ≤ 0.5, p value < 0.01). As early as three hours post inoculation (hpi), three of these genes were up-regulated: WRKY53 (AT4G23810, 2.47 fold), WRKY40 (2.22 fold), and NAC102 (AT5G63790, 2.18 fold). WRKY53 was also up-regulated by the disarmed *A. tumefaciens* strain GV3101 (2.37 fold) 3 hpi. Six days post inoculation (dpi), the expression of six transcription factor genes was up- or down-regulated (S1 Table). In *Arabidopsis* crown gall material of *A. tumefaciens* strain C58, 141 transcription factor genes were transcriptionally changed compared to reference tissue 35 days post wounding (dpw). Amongst these, 74 genes were up-regulated, with 67 down-regulated (S1 Table) and all belong to various families including WRKYs, MYBs, DOFs, and NACs. The DNA binding elements and the microarray data both suggest that the MYB, DOF, WRKY, bHLH, ARR1 and ARF transcription factors are potential candidates for involvement in the regulation of *Ipt* expression, while ARR1 could regulate transcription of the *IaaH* or *IaaM* genes. The core promoter sequence elements could contribute to the basal expression of the three oncogenes in plant cells, whereas the binding sites for transcription factors might function in enhancing their transcription.

### WRKY18, WRKY40, WRKY60 and ARF5 activate the *Ipt* oncogene promoter

**Figure 2:**
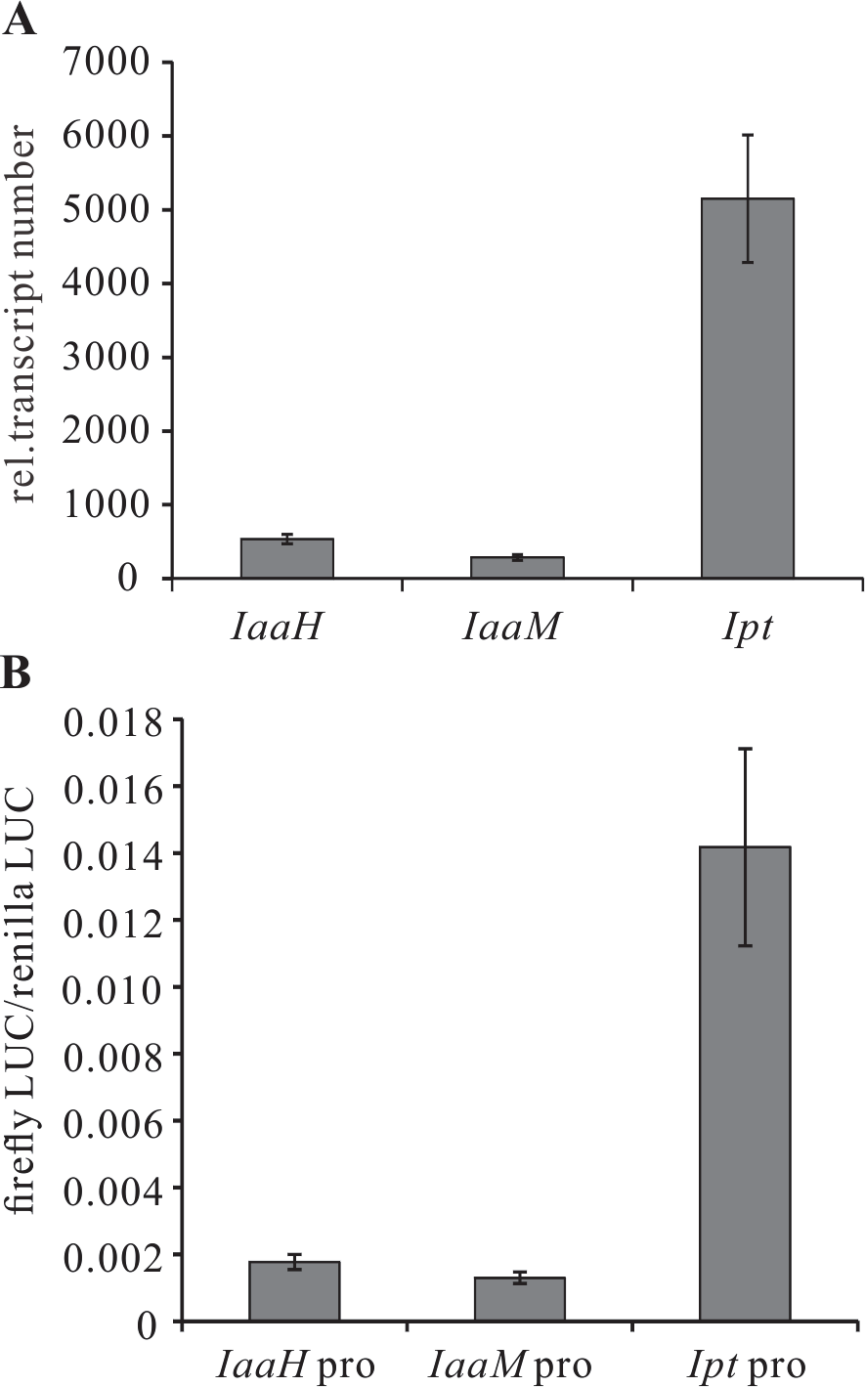
Transcripts of oncogenes in crown galls and activity of oncogene promoters in protoplasts. (A) Relative abundance of *IaaH, IaaM* and *Ipt* transcripts in crown gall tumors 25 days after inoculation of *A. tumefaciens* strain C58 into *Arabidopsis* inflorescence stems. Relative transcript numbers were quantified by qRT-PCR and normalized to 10,000 molecules of *ACTIN2/8*. Bars show mean values (±SD) of three independent samples. (B) Relative luciferase activity (firefly LUC/renilla LUC) driven by oncogene promoters (*IaaH* pro, *IaaM* pro and *Ipt* pro). Relative luciferase activity is calculated by firefly luminescence/renilla luminescence. Bars show mean values (±SD) of three independent experiments.

To begin to study the regulation of onocgene expression, we first used quantitative real-time PCR (qRT-PCR). We assessed the relative transcript numbers of *IaaH, IaaM* and *Ipt* genes in 25-day-old *Arabidopsis thaliana* crown galls induced by the virulent *A. tumefaciens* strain C58, finding that the transcript levels of *IaaH* and *IaaM* were much lower compared to those of the *Ipt* gene in the crown galls (Fig. 2A). The high-throughput protoplast transactivation (PTA) system was then used [48] to identify transcription factors that could activate the three oncogene promoters in plant cells. To do so, the complete promoters of *IaaH* (IGR1a, 337 bp), *IaaM* (IGR1b, 337 bp) and *Ipt* (IGR2, 697 bp) of the pTiC58-encoded oncogenes (Fig. 1A) were fused with the CDS of the firefly luciferase (LUC) reporter gene. The plasmids containing the oncogene promoter-LUC constructs were transfected into *Arabidopsis* mesophyll protoplasts, either alone, or together with a second plasmid containing the CDS of a transcription factor fused to the constitutive cauliflower mosaic virus (CaMV35S) promoter. The relative luminescence, a measure for the oncogene promoter activity since it drives luciferase gene expression, was then determined. Mesophyll protoplasts transfected only with the oncogene promoter-LUC constructs showed the same pattern of promoter activity as that determined for the relative transcript numbers in crown galls (Fig. 2B). The *Ipt* promoter induced a higher relative luminescence than the *IaaH* and *IaaM* promoters.

**Figure 3:**
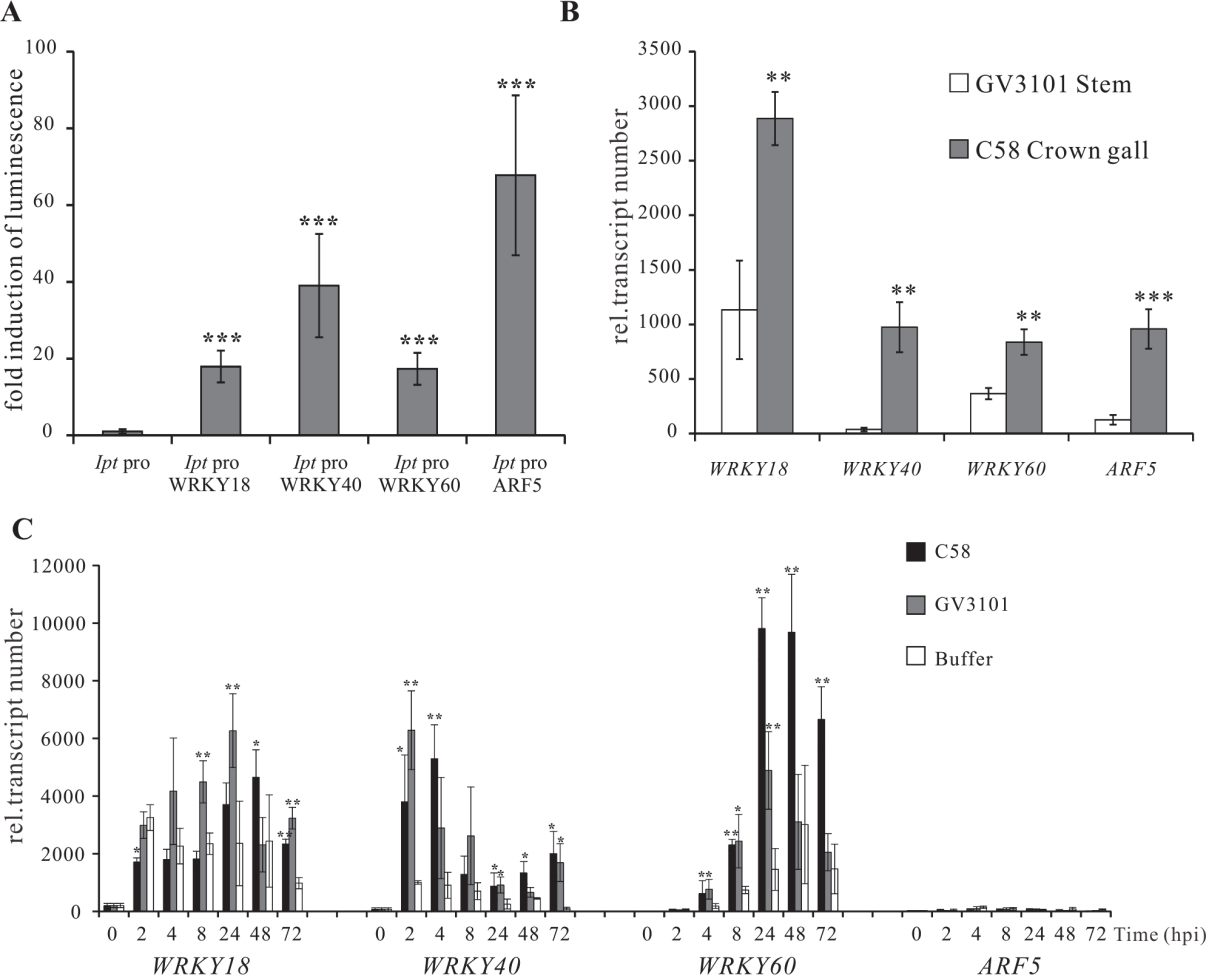
Activation of the *Ipt* promoter and gene expression of *WRKY18, WRKY40, WRKY60* and *ARF5*. (A) Fold induction of *Ipt* promoter-driven luminescence (*Ipt* pro) by WRKY18, WRKY40, WRKY60 and ARF5 in *Arabidopsis* mesophyll protoplasts transfected with two plasmid types. One harbors the *Ipt* promoter upstream of the firefly luciferase coding sequence (CDS) and the other, the universal cauliflower mosaic virus promoter (CaMV35S) upstream of a transcription factor CDS. The relative luminescence induced by the *Ipt* promoter in the absence of a transcription factor expression plasmid was set to 1. Bars show mean values (±SD) of three independent experiments. (B) Relative transcript numbers of *WRKY18, WRKY40, WRKY60* and *ARF5* genes in crown galls 25 days after inoculation with the virulent *A. tumefaciens* strain C58 (C58 Crown gall) and the disarmed strain (GV3101 Stems). (C) Time-dependent expression of the *WRKY18, WRKY40, WRKY60* and *ARF5* genes upon infiltration of five-week-old *Arabidopsis* leaves with suspension (OD_600_ 1.0) of strain C58 and GV3101 as well as an Agromix buffer as control. Relative transcript numbers were quantified by qRT-PCR and normalized to 10,000 molecules of *ACTIN2/8*. Bars show mean values (±SD) of three independent samples. ** *P<0.01* *** *P<0.001* (Student’s *t*-test).

Next, a library containing the CDS of more than 400 transcription factors was screened. Among the included family members, WRKY, AP2/ERF, bHLH, bZIP, DOF, MYB and NAC, only WRKY18 (AT4G31800), WRKY40, WRKY60 (AT2G25000) and ARF5 were found to specifically activate the *Ipt* promoter in protoplasts (Fig. 3A). Protoplasts co-transfected with the WRKY or ARF effector and the *Ipt*-promoter-LUC reporter constructs exhibited a significantly higher promoter activity (reflected by luciferase activity) compared to the control samples that only harbored the reporter. Despite several attempts, no transcription factor was found to activate the *IaaH* and *IaaM* promoters. Comparison of the three WRKYs alone and in combination both showed that WRKY40 exerts the strongest impact on *Ipt* promoter-driven luciferase expression (S4 Fig.). Even all three WRKYs together did not increase the relative luminescence more than WRKY40 alone. This observation points towards a dominant role for WRKY40 in *Ipt* promoter regulation.

The transcript numbers of *WRKY18, WRKY40, WRKY60* and *ARF5* genes in crown gall tissues of *A. tumefaciens* strain C58 were determined using qRT-PCR. In agreement with the published microarray data [20,46], the transcript levels were clearly elevated in crown gall tumors compared to inflorescence stems inoculated with the disarmed *A. tumefaciens* strain GV3101 (Fig. 3B). It is already known that *WRKY18, WRKY40* and *WRKY60* are induced early after bacterial and fungal pathogen infection [49,50]. To analyze the impact of *A. tumefaciens* on gene induction, we analyzed the time-dependent expression of the three *WRKY* genes in *Arabidopsis thaliana* (Col-0) leaf tissues infiltrated with either the virulent *A. tumefaciens* strain C58, the disarmed strain GV3101 or buffer as a control. The qRT-PCR results demonstrated that the three *WRKY* genes responded to a certain degree to the infiltrated buffer solution at all analyzed time points (2 hpi to 72 hpi), indicating that they respond to wounding (Fig. 3C). The transcript levels of *WRKY18* began to increase significantly at 8 hpi after infiltration by strain GV3101. The *WRKY40* and *WRKY60* genes were significantly induced by both *A. tumefaciens* strains as early as 2 and 4 hpi, respectively (Fig. 3C). In contrast, transcription of the *ARF5* gene was still very low after 72 hpi, suggesting that this gene is not responsive to *A. tumefaciens* or wounding at the time points analyzed (Fig. 3C). The gene expression patterns imply that at the very beginning of *A. tumefaciens* infection (2 to 4 hpi), *WRKY40* and *WRKY60* genes are already expressed.

WRKY and ARF transcription factors bind respectively to specific DNA sequences, W-box (TGAC) and AuxRE (TGTCNC or TGTCTN). Sequence analysis of the two IGRs of pTiC58 revealed that seven W-boxes (one W-box is localized in the 5’ UTR of the *Ipt* gene) and five AuxREs are located in IGR2 (Table 1, 2), which are equally distributed along the promoter sequence (S5 Fig.). IGR1 drives expression of *IaaH* and *IaaM* and contains only one W-box and AuxRE sequence motif, and this is more closely localized upstream of the *IaaM* than that of the *IaaH* TATA box. Sequence comparisons of IGR1 and IGR2 regions illustrate that W-boxes and AuxREs are also conserved in the T-DNA regions of several *A. tumefaciens* strains (Table 2). Similar to the pTiC58, the majority of these elements are enriched in the *Ipt* promoters whereas only one or two of them are located in the *IaaH* and *IaaM* promoter sequences. From this *in silico* result, it can be concluded that the *Ipt* oncogenes, rather than *IaaH* and *IaaM* of the different *A. tumefaciens* strains are regulated by WRKY and ARF transcription factors *in planta*.

**Table 2:** Number of WRKY-boxes (W-boxes) and auxin response elements (AuxREs) within the intergenic regions (IGRs) of the tumor inducing (Ti) plasmids from different *A. tumefaciens* strains.

		IGR1^a^		IGR2^b^	
	pTi plasmid	W-box (TGAC)	AuxRE (TGTCNC or TGTCTN)	W-box (TGAC)	AuxRE (TGTCNC or TGTCTN)
Nopaline	pTiC58	1	1	7	5
	pTiT37	?	?	5	5
	pTiSAKURA	2	1	6	5
Octopine	pTi15955	2	1	7	5
	pTiA6NC	2	1	7	5
	pTiAch5	2	1	7	5
Agropine	pTiBo542	2	1	6	3

Sequences are from the Genbank database (http://www.ncbi.nlm.nih.gov/genbank/). ”?”, IGR1 of pTiT37 is not present in the Genbank database.

^a^ IGR1 is localized between the coding sequences of *IaaH* and *IaaM*

^b^ IGR2 is localized between the coding sequences of*IaaM* and *Ipt.*

### WRKY18, WRKY40 and WRKY60 mutants display an impaired crown gall development

**Figure 4:**
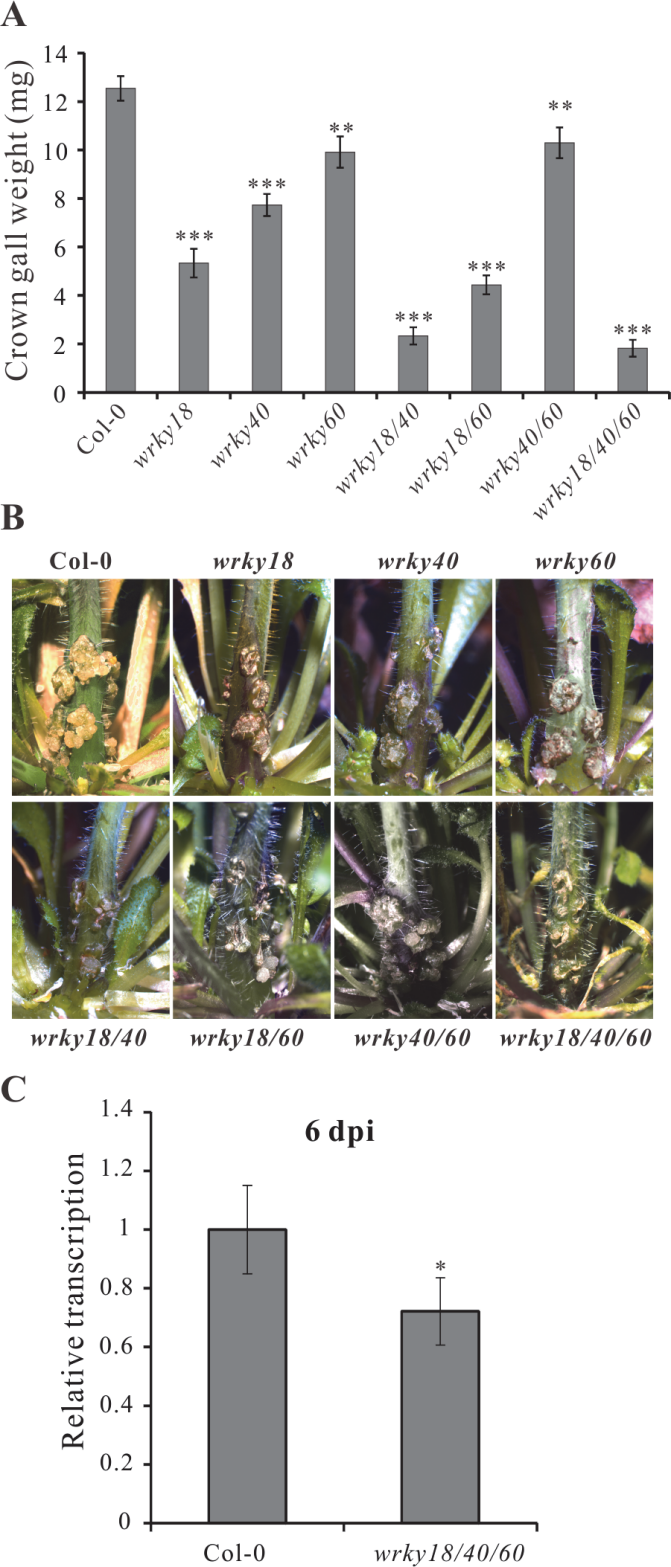
*Arabidopsis wrky* mutants develop smaller crown galls. (A) Crown gall weights of *wrky18, wrky40, wrky60* mutants and the wild type Col-0 25 days after inoculation of *Arabidopsis* inflorescence stems with the tumorigenic *A. tumefaciens* strain C58. Bars show mean values of crown gall weight (±SE) separated from the stems of at least 40 plants from each genotype. (B) Representative pictures of the stems of the different genotypes 25 days after inoculation of *A. tumefaciens*. (C) Relative transcript numbers of the *Ipt* oncogene in stems of the wild-type plant Col-0 and the *wrky18/40/60* triple mutant 6 days post inoculation (6 dpi) of *A. tumefaciens* strain C58. Relative transcript numbers were quantified by qRT-PCR and normalized to 10,000 molecules of *ACTIN2/8*. Bars show mean values (±SD) of three independent samples. * *P<0.05;* ** *P<0.01*; *** *P<0.001* (Student’s *t*-test).

To unravel the role of WRKY18, WRKY40 and WRKY60 in *A. tumefaciens*-mediated crown gall development, we performed a crown gall growth assay with mutant plants of the three *WRKY* genes inoculated with the tumorigenic *A. tumefaciens* strain C58, determining the crown gall weights 25 days later. All mutant genotypes developed smaller crown galls than the wild-type Col-0 (Fig. 4A, B), with the double mutant *wrky18/wrky40* and the triple mutant *wrky18/40/60* developing the smallest crown galls. The triple mutant was most resistant to crown gall development; about 30% of the mutant plants did not development any crown gall material at all after 25 days. Unfortunately, the role of the ARF5-mediated auxin signaling pathway on crown gall development could not be analyzed due to the strong developmental phenotypes of *arf5* mutant plants [51,52].

If WRKY18, WRKY40 and WRKY60 activate the *Ipt* promoter, it would be expected that *Ipt* oncogene expression would be altered in the WRKY mutant plants. To investigate this, we used quantitative RT-PCR to measure the relative transcript numbers of the *Ipt* oncogene in *Arabidopsis* crown gall material of the *wrky* mutants inoculated with *A. tumefaciens* strain C58. Compared to crown galls from the wild-type (Col-0) plants, the *Ipt* transcript levels were similar in crown galls from the *wrky18, wrky40* and *wrky60* mutants (S6A Fig.). Due to this similarity, i.e., no obvious impact of WRKY on long term *Ipt* gene expression in crown galls, earlier time points of C58 *Arabidopsis* stem inoculations were analyzed. At 2 dpi, the *Ipt* transcript levels were far too low to reliably quantify differences (S6B Fig.). Only at 6 dpi did *Ipt* transcription reach a measureable level (S6B Fig.) and showed in the triple mutant (*wrky18/40/60*) a moderate reduction compared to the wild-type (Fig. 4C). The moderate reduction of *Ipt* transcription may be due to the function of ARF5, which is still expressed in the *wrky* triple mutant. This assumption is supported by the observation that in crown galls of the *wrky* single mutants gene expression of ARF5 was elevated and that of IAA12, an inhibitor of ARF5 function, was reduced (S6C Fig.).

### WRKY40 and ARF5 proteins interact and synergistically potentiate *Ipt* promoter activity

**Figure 5:**
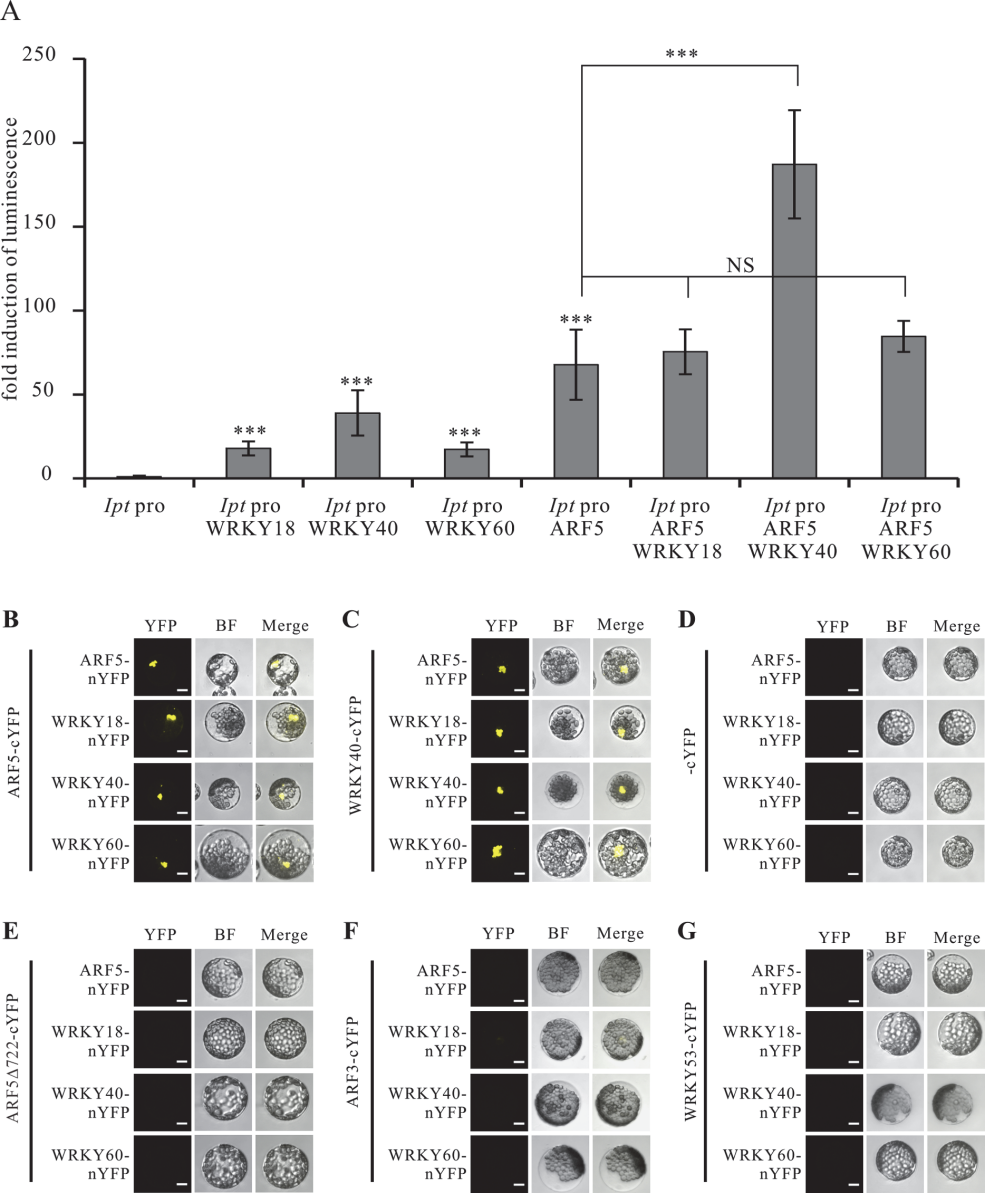
WRKY40 and ARF5 protein interaction potentiates *Ipt* promoter activity. (A) Fold induction of *Ipt* promoter-driven luminescence in the presence of WRKY18, WRKY40, WRKY60 and ARF5 transcription factor expression plasmids in the protoplast transactivation system. The relative luminescence induced by the *Ipt* promoter in protoplasts without transfection of any of the transcription factor expression plasmids was set to 1. Bars show mean values (±SD) of three independent experiments *** *P<0.01* (Student’s *t*-test). NS: not significant. (B) Bimolecular fluorescence (BiFC) assay with ARF5-cYFP and ARF5-nYFP, WRKY18-nYFP, WRKY40-nYFP, WRKY60-nYFP, (C) with WRKY40-cYFP, (D) with cYFP, (E) with a C-terminal deletion of ARF5 (ARF5Δ722-cYFP), (F) with ARF3-cYFP and (G) with WRKY53-cYFP in *Arabidopsis* mesophyll protoplasts. YFP, image in fluorescence mode of reconstituted yellow fluorescent proteins; BF, images in bright filed mode; merge, overlay of YFP with the corresponding BF image. Bars, 10 μm.

The PTA data revealed that the *Ipt* promoter can be activated by WRKY18, WRKY40, WRKY60 and ARF5. To test whether these transcription factors cooperatively regulate the *Ipt* promoter, we co-expressed the WRKY40 protein with ARF5 in the presence of the *Ipt* promoter-LUC construct in *Arabidopsis* mesophyll protoplasts. The *Ipt* promoter-driven luciferase activity was clearly higher, particularly in the presence of ARF5 and WRKY40 compared to ARF5 or WRKY40 alone (Fig. 5A). In contrast, expression of ARF5 together with WRKY18 or WRKY60 did not further enhance the *Ipt* promoter activity. This also indicates that WRKY40 is more important than WRKY18 and WRKY60 for activating the *Ipt* promoter.

These results imply that the WRKY40 and ARF5 proteins interact to synergistically activate *Ipt* gene expression. This was tested using the Bimolecular Fluorescence Complementation (BiFC) assay to study protein interactions between the WRKYs and ARF5. The C-terminal half of the yellow fluorescent protein (cYFP) was fused to the C-terminus of the ARF5 and WRKY40 proteins to express ARF5- and WRKY40-cYFP fusion proteins, respectively. The N-terminal half of YFP (nYFP) was fused to the C-terminus of the three WRKY proteins as well as to ARF5 to generate WRKY18-, WRKY40-, WRKY60-nYFP and ARF5-nYFP. Observation of YFP-mediated fluorescence demonstrates that both WRKY40 and ARF5 interacted with themselves and with all the other expressed genes, when transiently co-expressed in *Arabidopsis* mesophyll protoplasts (Fig. 5B, C). The fluorescence signal was always restricted to the nucleus. The free cYFP construct was used as negative control, and showed no YFP fluorescence when co-expressed with the WRKY-nYFPs and ARF5-nYFP in protoplasts (Fig. 5D).

It has been reported that the domain III and IV at the C-terminus of the ARF5 protein is important for dimerization and protein-protein-interaction [53–55]. To prove whether these domains are required for the interaction with the WRKY proteins, we fused a C-terminal deletion of ARF5 (1–722 aa) to cYFP (ARF5Δ722-cYFP) and co-expressed them with either ARF5-nYFP or the three WRKY-nYFPs. Although stable [53], the truncated ARF5Δ722 protein was unable to interact with the intact ARF5 protein or with WRKY18, WRKY40 and WRKY60 (Fig. 5E). This indicates that the domains III and IV are not only required for self-interaction, but also for interaction with the three WRKYs. The specificity of the interactions between ARF5 and the three WRKYs was confirmed by co-expressing ARF3 (AT2G33860)-cYFP, which naturally lacks domain III and IV, and WRKY53-cYFP, expressed early after infection with *A. tumefaciens* strain C58 (3 hpi; S2 Table) [20,53]. Neither ARF3 nor WRKY53 interacted with ARF5, WRKY18, WRKY40, and WRKY60, thus verifying that the interactions between the WRKYs and ARF5 are specific (Fig. 5F, G).

**Figure 6:**
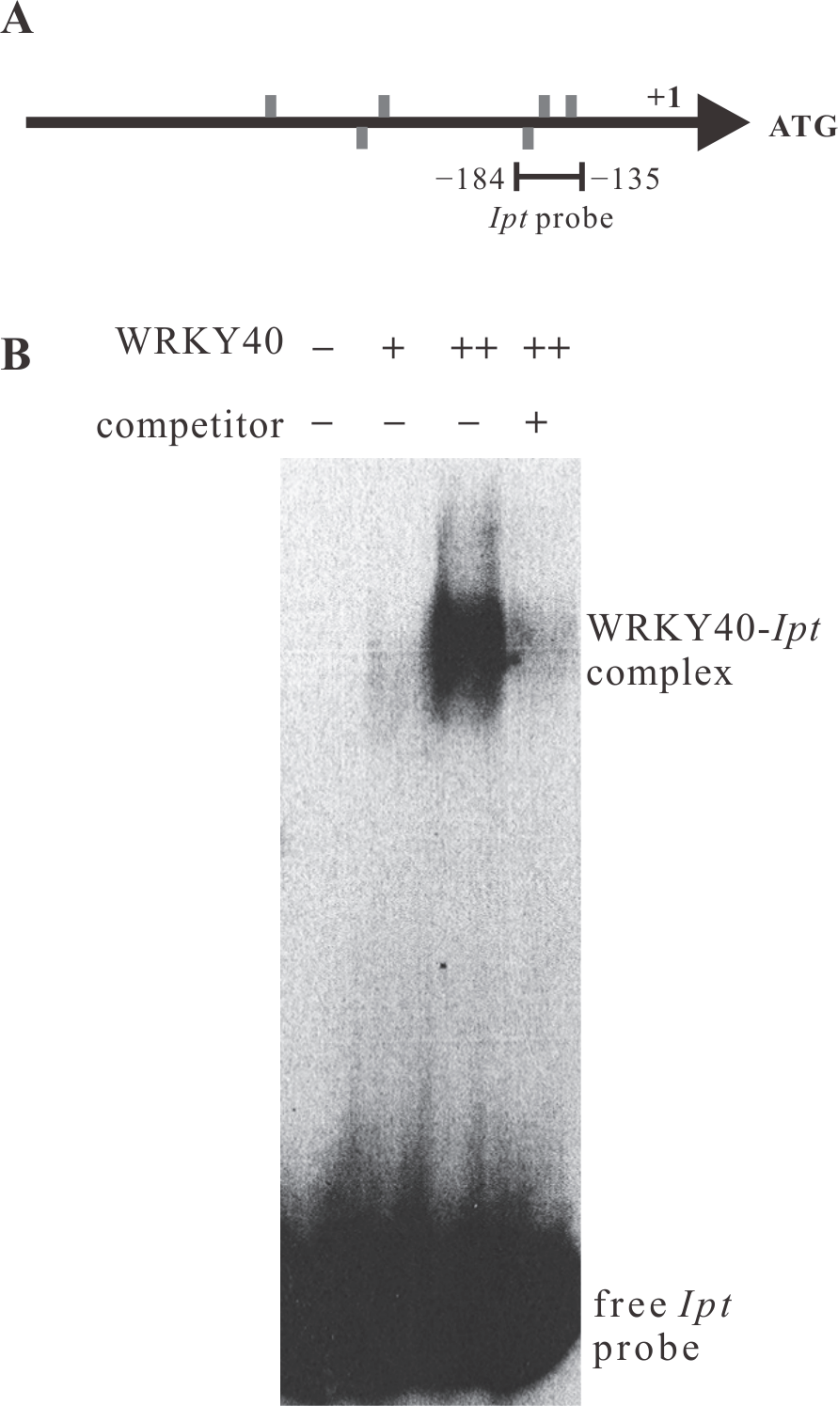
WRKY40 binds to the *Ipt* promoter *in vitro*. (A) Positions of W-boxes (TGAC, grey bars) in the sense (above the line) and anti-sense strand (below the line) of the *Ipt* promoter. The line below the *Ipt* promoter (−184 bp to −135 bp) indicates the fragment used as *Ipt* probe for electrophoretic mobility shift assay (EMSA). (B) EMSA with the labeled *Ipt* promoter probe in the absence (−) and in the presence of 150 ng (+) or 300 ng (++) of purified recombinant histidine-tagged WRKY40 protein. Competitor indicates without (−) and with (+) addition of unlabeled *Ipt* promoter probe. The WRKY40-*Ipt* complex indicates binding of WRKY40 protein to the labeled *Ipt* probe and the free *Ipt* probe no protein binding.

The PTA assays indicate that WRKY40 has a stronger potential to activate the *Ipt* promoter than WRKY18 and WRKY60 (Fig. 3A and 5A). This implies that WRKY40 regulates the *Ipt* promoter directly. We therefore analyzed binding of WRKY40 to the *Ipt* promoter using the electrophoretic mobility shift assay (EMSA). The recombinant WRKY40 protein fused to six histidine amino acids at the N-terminus (6×His-WRKY40) was expressed and purified from *E. coli* and a 50 bp fragment (−184 bp to −135 bp) of the *Ipt* promoter, which contains three of the six W-boxes located in the promoter region, was radioactively labeled and served as a probe for EMSA (Fig. 6A). Only a weak band of the shifted *Ipt* promoter fragment (Fig. 6B, WRKY40-*Ipt* complex) was observed in the presence of 150 ng purified recombinant 6×His-WRKY40 protein, but a doubled amount of the His-tagged WRKY40 protein (300 ng) exhibited a much stronger band. Addition of unlabeled *Ipt* promoter fragments as competitor to the reaction mixture significantly reduced the binding of WRKY40 to the labeled *Ipt* promoter probe. Thus, the WRKY40 protein binds to the *Ipt* probe *in vitro*, suggesting that the *Ipt* promoter is a direct target of the WRKY40 transcription factor in plant cells.

### The ARF5-mediated auxin-signaling pathway induces *Ipt*, but not *IaaH* and *IaaM* gene expression

That ARF5 enhances the WRKY40-mediated activation of the *Ipt* promoter suggests that the auxin signaling pathway is involved in regulating *Ipt* expression. Previous studies have shown that the levels of unconjugated IAA in infected *Arabidopsis* stems are more than two-fold higher six days after inoculation with *A. tumefaciens* strain C58 compared to non-inoculated plant stems [20]. We found that crown galls accumulate four times more unconjugated IAA than control tissues and the total level of cytokinins in *Arabidopsis* crown gall tissues infected with *A. tumefaciens* strain C58 are 10 times higher than in crown gall-free stem tissues (8414 vs. 849 ng/g dry weight). The dominant cytokinin forms in *Arabidopsis* crown gall tissues were zeatin conjugates, including zeatin nucleotide (3657 vs. 308 ng/g dry weight) and zeatin riboside (2294 vs. 76 ng/g dry weight). The content of free zeatin was also higher in crown gall tissues than in mock-inoculated stems (544 vs. 34 ng/g dry weight).

**Figure 7:**
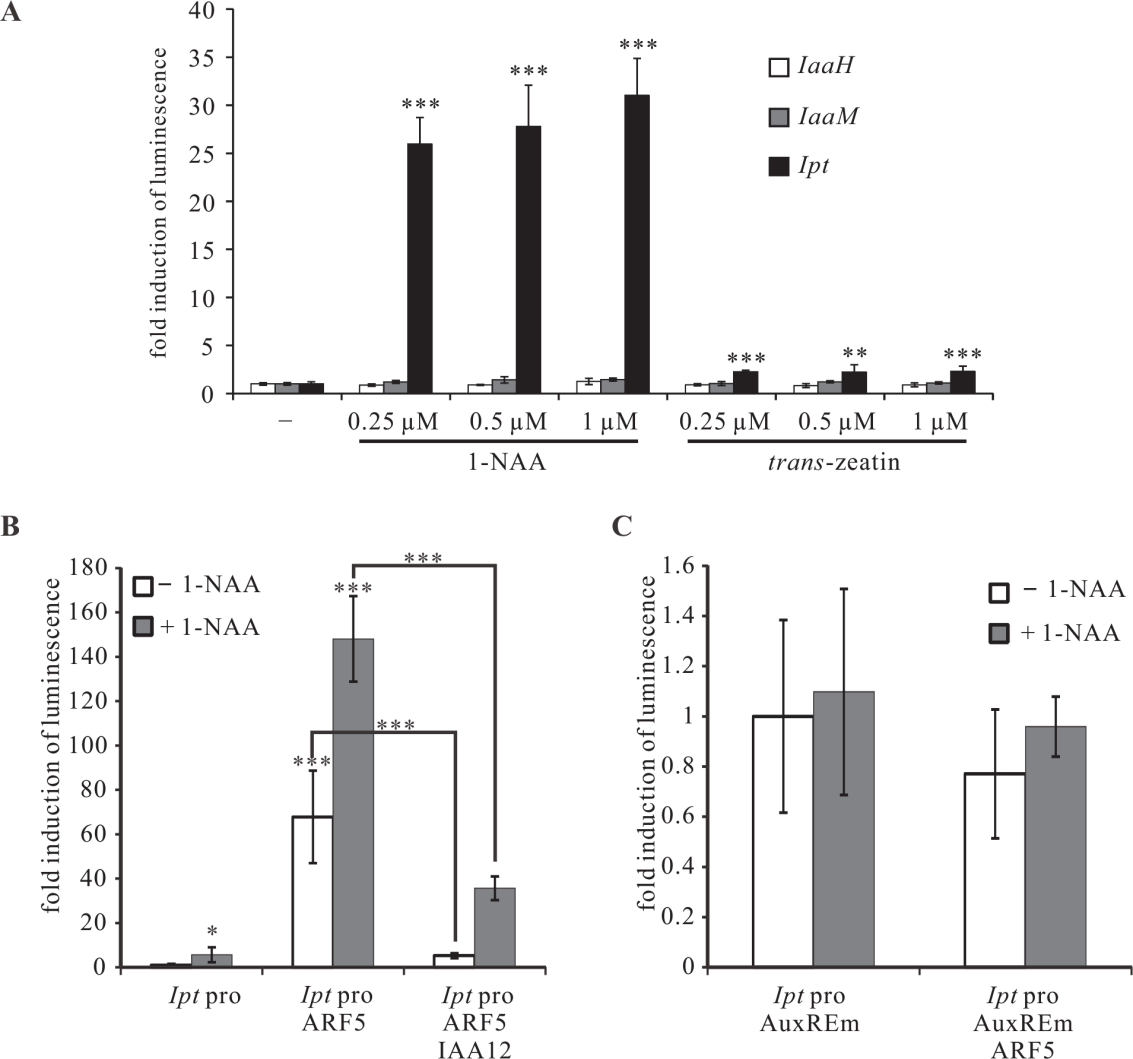
ARF5 activates the *Ipt* promoter in an auxin-dependent manner. (A) Fold induction of oncogene promoter-driven luminescence in *Arabidopsis* mesophyll protoplasts treated with auxin (1-NAA) or cytokinin (*trans*-zeatin) of different concentrations. Protoplasts were transfected with the *IaaH, IaaM*, and *Ipt* promoter-luciferase reporter constructs, and then incubated with different concentrations of 1-NAA or *trans*-zeatin overnight. (B) Fold induction of *Ipt* promoter-driven luminescence in *Arabidopsis* mesophyll protoplasts (*Ipt* pro) in the presence of the transcription factor expressing plasmids ARF5 (*Ipt* pro ARF5) or ARF5 plus IAA12 (*Ipt* pro ARF5 IAA12) with (+ 1-NAA) and without auxin (− 1-NAA) addition. (C) Mutations in the five auxin responsive elements (AuxREm, TGTCNC to TGGCNC and TGTCTN to TGGCTN) of the *Ipt* promoter abolish the ARF5- and auxin-dependent luminescence induction. The relative luminescence of intact or mutated *Ipt* promoters in the absence of any transcription factor expression plasmids and auxin treatment was set to 1. Bars show mean values (±SD) of three independent experiments. * *P<0.05*; ** *P<0.01*; *** *P<0.001* (Student’s *t*-test).

Based on these results, we used the PTA system to analyze the impact of auxin and cytokinin on *IaaH, IaaM* and *Ipt* promoter activity. The *Ipt* promoter was highly activated by the bioactive auxin type 1-naphthaleneacetic acid (1-NAA) and the cytokinin type *trans*-zeatin (Fig. 7A), with the latter much less effective. Increasing concentrations of auxin and cytokinin had no strong enhancing effect on the activity of the three oncogene promoters (Fig. 7A). The *Ipt* promoter sequence contains five auxin response elements (AuxREs, TGTCNC or TGTCTN) for binding of ARF transcription factors, whereas only one AuxRE is present in the bidirectional *IaaH* and *IaaM* promoter sequence (Table 1, S5 Fig.) and ARF transcription factors usually regulate their target genes in an auxin-dependent manner [33,34]. Thus, we analyzed the regulatory effect of ARF5 on the *Ipt* promoter in the presence of auxin in the PTA system. ARF5 activated the *Ipt* promoter, an activation that was even stronger when the mesophyll protoplasts were treated with auxin (1-NAA, Fig. 7B). Mutations in the AuxREs (sense TGTCNC or TGTCTN, anti-sense GNGACA or NAGACA) in the *Ipt* promoter abolished the auxin induction and the enhancing effect of ARF5 (Fig. 7C). It is known that auxin/indole-3-acetic acid (Aux/IAA) proteins can inhibit ARF mediated promoter activation and the repressor of ARF5 is IAA12 (also known as BODENLOS, BDL, AT1G04550) [54]. When we co-transfected *Arabidopsis* mesophyll protoplasts with the ARF5 and IAA12 plasmid constructs, a significant reduction in the *Ipt* promoter-driven luciferase activity was found compared to protoplasts transfected with only ARF5 (Fig. 7B). Nonetheless, the level of the *Ipt* promoter activity was not as low as it was in the absence of any transcription factor, indicating that not all ARF5 proteins are inhibited by IAA12.

In addition to the W-boxes and AuxREs, the *IaaH, IaaM* and *Ipt* promoters also contain ARR1 binding elements (GATT; Table 1), suggesting that the three oncogenes are regulated by type-B ARR transcription factors to mediate cytokinin signaling. The ARR1 gene is expressed at low levels in crown gall tissue of the virulent *A. tumefaciens* strain C58 and in stems infected with the disarmed strain GV3101 according to real time PCR measurements (S7A Fig.). *ARR4* (AT1G10470), a type A transcription factor gene, was strongly expressed in crown gall tumors (S7A Fig.). The ability of both the ARR1 and ARR4 transcription factors to activate the *IaaH, IaaM* and *Ipt* promoters was tested in the PTA system. Neither ARR1 nor ARR4 significantly increased luciferase activity driven by the three oncogene promoters, even in the presence of *trans*-zeatin (S7B Fig.). Hence, the ARF5-mediated auxin signaling pathway, but not that of cytokinin, regulates *Ipt* expression, whereas the expression of *IaaH* and *IaaM* is not affected by either of the two signaling pathways.

## Discussion

The plant pathogen, *Agrobacterium tumefaciens* takes advantage of the host transcriptional machinery to express its own T-DNA encoded oncogenes *IaaH, IaaM* and *Ipt* in plant cells. Expression of the oncogenes results in increased production of the phytohormones auxin and cytokinin, which induce uncontrolled cell proliferation and crown gall development. The T-DNA transformation process and the roles of the encoded oncogene enzymes is far better understood [10,56,57] than the regulation of oncogene expression in plant cells. We therefore examined this regulation, asking whether the expression of the *IaaH, IaaM* and *Ipt* oncogenes is regulated by host transcription factors and how oncogene expression is coordinated to obtain tumor-inducing auxin/cytokinin levels in a T-DNA transformed cell.

### *Agrobacterium tumefaciens* utilizes a transcription factor of the pathogen defense pathway to induce *Ipt* oncogene expression

Expression of a gene in a eukaryotic cell requires general sequence features (e.g. TATA, CAAT) and potentially *cis*-regulatory elements for the binding of transcription factors. For the *Ipt* promoter of the octopine Ti plasmid pTiAch5, previous studies have shown that it binds CBF, a protein of unknown function from tobacco nuclear protein extracts [25–27]. This implies that at the least, expression of the *Ipt* oncogene is regulated by plant derived transcription factors. Nonetheless, using the PTA screening system we found that no transcription factor activated the *IaaH* and *IaaM* promoters of pTiC58. This may be because the transcription factor collection used for screening did not cover all the encoded *Arabidopsis* transcription factors; candidates for binding to the *IaaH* and *IaaM* promoters may have been missed. However, the very few *cis*-regulatory elements in the relatively short promoter sequence and the low level of transcription in crown galls, in addition to the low promoter activity in protoplasts, suggest that the *IaaH* and *IaaM* genes are not strongly activated by transcription factors, but instead are constitutively expressed at low basal levels. In contrast, the *Ipt* oncogene promoter of pTiC58 contains several W-boxes and is activated by the *WRKY18, WRKY40* and *WRKY60* proteins. The impaired crown gall growth on the *wrky18, wrky40* and *wrky60* mutant plants indicates that these WRKY transcription factors have a positive effect on crown gall development. The three WRKYs are paralogous transcription factors that cooperatively regulate biotic and abiotic stress responses in *Arabidopsis* [49,58–63] and the respective *wrky* mutants are known to be more resistant to biotrophic pathogens such as *Pseudomonas syringae* and powdery mildew *Golovinomyces orontii* [50]. Hence, the smaller crown galls on these *wrky* mutants may result from both fewer transformation events due to the stronger resistance response towards biotrophic pathogens and/or from reduced *Ipt* expression due to the loss of *wrky* function. Unfortunately, these two processes are not easy to separate in infection-based assays.

It is known that transcription of *WRKY40* and *WRKY60* is induced by fungal and bacterial pathogens [49,50]. Likewise, *A. tumefaciens* inoculation induced their transcription within two hours, indicating that they are expressed quite early in response to this pathogen. Thus, it is conceivable that the WRKYs are needed to trigger *Ipt* oncogene expression from the very beginning in a T-DNA transformed cell, so these pathogen responsive genes are already expressed when the T-DNA enters the host cell. Consequently, a reduction in *Ipt* promoter activity can be observed early on in the *wrky* triple mutant, vanishing at later infection stages. The relatively moderate difference in *Ipt* gene expression between the *wrky* triple mutant and wild-type most likely results from the increased expression of *ARF5* and reduced expression of its inhibitor *IAA12* in the mutant background. Thus, *A. tumefaciens* hijacks a host transcription factor, which is part of the plant pathogen defense machinery, to initiate expression of its own oncogene in the host cell.

### Auxin, but not cytokinin signaling is important for inducing *Ipt* oncogene expression

*A. tumefaciens* and T-DNA transformed plant cells produce auxin and cytokinin [13,20]. Cytokinin affects cell division, essential for cell proliferation and initiation of crown gall development. Only the activity of the *Ipt* promoter, not that of the *IaaH* and *IaaM* genes, increased upon application of *trans*-zeatin, the dominant cytokinin in *Arabidopsis* crown galls. Eight binding elements for the ARR1 transcription factor are located in the bidirectional promoter of *IaaH*/*IaaM* and seven in the *Ipt* promoter. ARR1 is a type-B ARR transcription factor that activates transcription of cytokinin responsive genes [64,65]. Nonetheless, the activity of all three oncogene promoters was not influenced either by ARR1 or ARR4, even in the presence of *trans*-zeatin. This indicates that cytokinin signaling does not have a dominant role in oncogene expression. The auxin type 1-NAA was much more effective than *trans*-zeatin in activating the *Ipt* promoter, but again, not for the promoters of *IaaH* or *IaaM*. Elevated levels of free IAA are detectable in infected tissues six days after inoculation with *A. tumefaciens* strain C58 [20] and at the same infection stage, expression of the *ARF5* gene begins to increase, as shown in the microarray data (1.49 fold, *P value* = 0.006) [20,46]. The *Ipt* promoter contains five AuxREs and is activated by 1-NAA and by the auxin response factor ARF5 upon release from inhibition by IAA12 in an auxin-dependent manner. Expression of the *ARF5* gene is induced by auxin [66] and the elevated auxin levels in plant tissues infected and T-DNA transformed by *A. tumefaciens* most likely induce *ARF5* gene expression and de-repress the ARF5 protein by proteolysis of IAA12. The release of ARF5 inhibition in the presence of auxin leads finally to activation of the *Ipt* promoter in the T-DNA transformed plant cell and may contribute to the moderate differences of *Ipt* transcript numbers in the *wrky* mutants and wild-type. Taken together, the results indicate that auxin is an important factor in regulating *Ipt* oncogene expression, which exerts its function through the auxin-sensitive transcription factor ARF5.

### WRKY40 and ARF5 synergistically boost *Ipt* gene expression, thereby integrating host pathogen defense and auxin signaling

Our study shows that WRKY40 binds directly to the *Ipt* promoter *in vitro* and has the strongest effect on *Ipt* promoter activation in plant cells, an activation that increases even further in the presence of the ARF5 transcription factor. It has been shown that WRKY transcription factors specifically interact with different kinds of proteins [67] and WRKY18, WRKY40 and WRKY60 interact with each other and themselves [49], a result confirmed in this study. Moreover, WRKY18, WRKY40, and WRKY60 interact with ARF5. Most ARFs contain four important domains, except for ARF3, ARF13 and ARF17, which lack domain III and IV and ARF23, which has only domain I [31]. Domain III and IV are localized at the C-terminus of ARF proteins and are important for dimerization and interaction with Aux/IAA proteins [53]. According to our study, the domain III and IV of ARF5 seem to be required for the interaction with the three WRKY transcription factors. The interaction of ARF5 with WRKY40, but not that with WRKY18 and WRKY60, greatly enhances the activation of the *Ipt* promoter, so emphasizing the role of WRKY40 as the most important transcriptional activator of *Ipt* gene expression. Moreover, the WRKY40-ARF5 interaction links two signaling pathways for the regulation of *Ipt* gene expression: the ARF5-dependent auxin and WRKY-mediated pathogen defense pathway. Both pathways are activated in the host plant upon infection with *A. tumefaciens* and synergistically boost expression of the *Ipt* gene in T-DNA transformed cells.

### Conclusion

**Figure 8:**
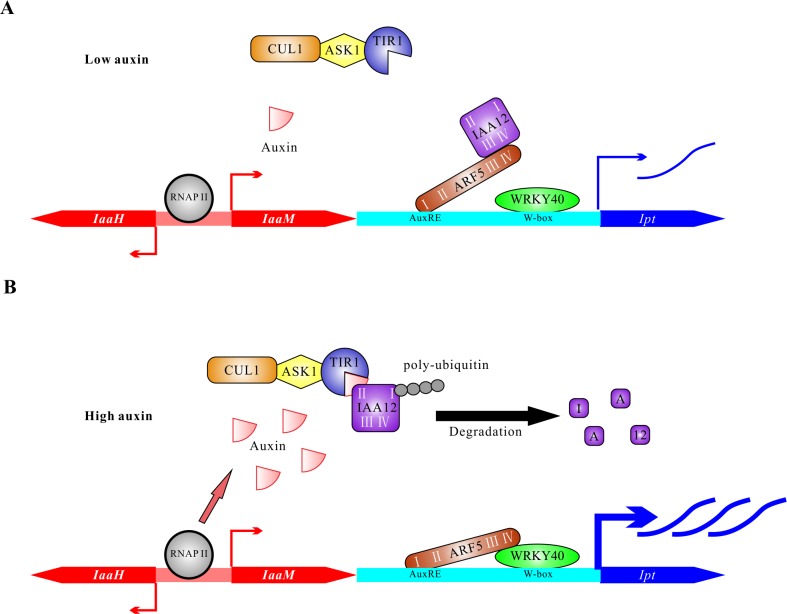
Proposed model on the regulation of oncogene expression in host cells. (A) The plant cells have low auxin levels at the early stage of transformation by *A. tumefaciens*. The bidirectional promoter between CDSs of *IaaH* and *IaaM* is recognized by RNA polymerase II complex (RNAP II) to induce basal levels of transcription. Under low auxin conditions, the transcriptional activity of ARF5 is largely blocked by IAA12 via the interaction of domain III and IV. The transcription factor WRKY40 binds to W-boxes (TGAC) located in the *Ipt* promoter and activates transcription of the *Ipt* gene. (B) T-DNA transformed plant cells contain elevated auxin levels six days after inoculation due to the constitutive activity of the *IaaH* and *IaaM* enzymes and secretion by *A. tumefaciens* cells. High concentration of auxin mediates the interaction between TIR1 and IAA12. Poly-ubiquitination of IAA12 by the complex of TIR1, SKP (ASK1) and cullin1 (CUL1) induces its degradation and releases ARF5 to bind to the AuxREs (TGTCNC or TGTCTN) in the *Ipt* promoter and form a complex with WRKY40 via domain III and IV. The ARF5-WRKY40 complex finally potentiates activation of the *Ipt* promoter and promotes expression of the *Ipt* oncogene.

This study suggests a bifactorial regulation of oncogene expression in T-DNA transformed plant host cells (Fig. 8). Just after *A. tumefaciens* infection, auxin and cytokinin levels are as low as in an untransformed plant cell. The WRKY40 gene is soon expressed in response to infection, and the protein binds to W-boxes in the *Ipt* promoter to induce gene expression (Fig. 8A). Under low auxin conditions, ARF5 interacts with IAA12, so is inactivated. Over time, the auxin concentration increases in the T-DNA transformed cell, the result of the ubiquitous expression of *IaaH* and *IaaM*, driven by binding the RNA polymerase II complex to the promoter and additional auxin that can be secreted from the *A. tumefaciens* cells into the apoplast. Under high auxin concentrations, the ARF5 inhibitor IAA12 is poly-ubiquitinylated and degraded, thus releasing the transcription factor ARF5. The de-repressed ARF interacts via domain III and IV with WRKY40, resulting in strong expression of the *Ipt* oncogene. Taken together, this transcription factor interaction integrates two signaling pathways: the WRKY-based pathogen defense pathway for initial induction of *Ipt* gene expression and later, the auxin signaling pathway to boost *Ipt* expression. Moreover, the alterations in *Ipt* expression levels may be a mechanism to fine-tune the cytokinin to auxin ratios in a transformed plant cell. The appropriate auxin/cytokinin balance is an important mechanism to control whether a crown gall will proliferate or grow and differentiate.

## Materials and Methods

### Plant materials and growth conditions

*Arabidopsis thaliana* ecotype Columbia (Col-0) was used as the genetic background of the *wrky18* (GABI-Kat 328G03)*, wrky40* (SLAT_N40001), and *wrky60* (SALK_120706) mutants [58]. Plants were grown on soil and cultivated in growth chambers (Percival AR-66L2, Perry, USA) with 12 h light (*ca*. 120 μmol·m^−2^·s^−1^ fluorescent white light, TL70, Philips, Eindhoven, Netherlands) at 22°C and 12 h dark at 16°C. The crown gall callus cell culture was generated by inoculating *A. thaliana* root segments of ecotype Wassilewskija (WS-2) with the virulent *Agrobacterium tumefaciens* strain C58 and cultivated on MS agar plates [1× MS basal salts including vitamins and MES buffer (Murashige and Skoog medium, Duchefa Biochemie, Haarlem, Netherlands), 10 g/L sucrose, 100 mg/L myo-Inositol (Duchefa Biochemie, Haarlem, Netherlands), 7.5 g/L plant agar (Duchefa Biochemie, Haarlem, Netherlands), pH 5.7] without the addition of phytohormones, but with 100 mg/L ticarcillin disodium/clavulanate potassium (Duchefa Biochemie, Haarlem, Netherlands). The GFP expressing crown gall cell cultures were generated in the same manner except the *A. tumefaciens* strain C58 was used. This harbored, in addition to its pTiC58 plasmid, the binary vector pMDC206 [68] with the *IaaH, IaaM* and *Ipt* promoter-green fluorescent protein (GFP) constructs inserted in the T-DNA region. The antibiotic hygromycin (30 mg/L) was added to the agar medium for selection of transformed cells. All callus cultures were transferred to fresh media every three weeks. The crown gall cell suspension cell cultures were grown in the dark at 22°C with gentle shaking at 160 rpm, and transferred to fresh medium [1× MS basal salts including vitamins and MES buffer (Murashige and Skoog medium, Duchefa Biochemie, Haarlem, Netherlands), 20 g/L sucrose, 100 mg/L myo-Inositol (Duchefa Biochemie, Haarlem, Netherlands), pH 5.7] at a 1:2 dilution (v/v) twice a week.

### *Agrobacterium tumefaciens* strains, cultivation and plant inoculation procedures

The virulent *A. tumefaciens* strain C58 noc^c^ (nopaline catabolism, number 584; Max Planck Institute for Plant Breeding, Cologne, Germany) and the disarmed derivative of C58, strain GV3101 (pMP90) were used for plant inoculations. The strains were cultivated on YEB-agar plates (5 g/L yeast extract, 5 g/L tryptone, 5 g/L sucrose, 50mM MgSO_4_, and 15 g/L agar) at 28°C for 2 days. GV3101 was cultivated in the presence of rifampicin (10 mg/L) and gentamicin (25 mg/L). Before plant inoculation, the *A. tumefaciens* strains were transferred into King’s liquid medium (20 g/L protease peptone, 1.5 g/L K_2_HPO_4_, 10 mL/L glycerol, 600 μM MgSO_4_) and grown overnight at 28°C and 140 rpm. King’s medium was removed by pelleting the bacteria three times at 8000 rpm for 1 min and resuspension in Agromix buffer (0.01 M MgCl_2_, 0.01 M MES pH 5.6). For recovery, the resuspended cells were cultured at 28°C and 140 rpm for 2 to 3 hours. The optical density (OD_600_) was measured at 600 nm (NanoDrop 2000c UV-Vis Spectrophotometer, Thermo, Waltham, USA) and adjusted to OD_600_ 1.0 for leaf infiltrations and OD_600_ 0.5 for inflorescence stem inoculations. *A. tumefaciens* suspensions were infiltrated into the abaxial side of 5-week-old *Arabidopsis* (Col-0) leaves by tightly pressing the orifice of a 1 mL syringe onto the leaf surface. For induction of crown gall growth, young inflorescence stems (3 to 10 cm) of *A. thaliana* plants were inoculated by injecting *A. tumefaciens* suspensions four times with a 5 mL syringe and a needle attached to it. Crown galls were separated from the inflorescence stems 25 days after inoculation with a scalpel using a dissecting microscope (Leica MZ6) and their weight was immediately determined. Leaves infiltrated or stems inoculated with *A. tumefaciens* strain GV3101 served as reference.

### Construction of recombinant plasmids

For construction of the promoter-GFP fusions (*IGR1a::GFP, IGR1b::GFP and IGR2::GFP*), the vector pMDC206 was used, which contains the coding sequence (CDS) of GFP including an intron [68]. The promoter sequences of the intergenic regions (Fig. 2A) between the *IaaH* and *IaaM* CDS (IGR1, 337 bp) and between the *IaaM* and *Ipt* CDS (IGR2, 697 bp) of the pTiC58 plasmid were inserted upstream of the GFP CDS using Gateway cloning technology [68]. IGR1 was cloned in both directions (IGR1a and IGR1b, Fig. 2B). The ubiquitous cauliflower mosaic virus (2× CaMV35S) promoter was used as a positive control. To construct the plasmids for the Bimolecular Fluorescence Complementation (BiFC) assay and the luciferase reporter constructs, the pSAT vector was altered to be used in the USER cloning strategy as described in [69,70]. For the BiFC assay, the ubiquitin 10 (UBQ10) promoter and CDS of the C-terminal half (Venus, 156–239) and N-terminal half (Venus, 1–173aa, I152L) of the yellow fluorescent protein (cYFP, nYFP) were inserted into the pSAT vector. The full CDSs, excluding the stop codon of WRKY18, WRKY40, WRKY53, WRKY60, ARF3 and ARF5, and of the C-terminal deletion of ARF5 (1–722 aa), were inserted before the C-terminus of the cYFP or nYFP to generate the fusion proteins WRKY-cYFP, WRKY-nYFP, ARF-cYFP, ARF-nYFP and ARF5Δ722-cYFP. To generate the *IaaH, IaaM, Ipt* promoter-firefly luciferase reporter constructs (*IaaH* promoter-LUC, *IaaM* promoter-LUC and *Ipt* promoter-LUC), DNA fragments of the luciferase reporter CDS and the CaMV-terminator were introduced into the pSAT vector first, then the sequences of IGR1a, IGR1b and IGR2 (Fig. 2B) were added upstream of the luciferase reporter CDS. To express a histidine-tagged WRKY40 protein in *E. coli* cells, full length CDS including the stop codon was cloned into the vector pET28b (Novagen Merck Millipore, Darmstadt, Germany) at the *Nde*I and *Xho*I restriction enzymes sites. This resulted in expression of a WRKY40 protein fused at its N-terminus with 6× histidine amino acids (6×His-WRKY40). For site-specific mutagenesis of the AuxREs in the *Ipt* promoter (*Ipt* promoter AuxREm), the QuickChange Site-Directed Mutagenesis Kit (Agilent Technologies, Santa Clara, USA) was used. All primer sequences used are listed in S2 Table.

### 5’ rapid amplification of cDNA ends (5’ RACE)

For analysis of the transcription start sites of the *IaaH, IaaM* and *Ipt* oncogenes of *A. tumefaciens* strain C58 in plant cells, the mRNA extracted from crown gall callus cells was used. The mRNA was extracted from approximately 50 mg crown gall callus material by using Dynabeads Oligo (dT)_25_ (Invitrogen, Carlsbad, USA) following the manufacturer’s protocol. First-strand cDNA was generated by using SMARTScribe Reverse Transcriptase, the SMARTer II A Oligonucleotide primer and the 5’ RACE CDS primer A (Clontech, Otsu, Japan). The fragments of the 5’ ends of the oncogene cDNAs were amplified using DreamTaq DNA Polymerase (Fermentas, Thermo, Waltham, USA) and the Universal Primer A Mix (UPM) and the gene specific primers (*IaaH* reverse, *IaaM* reverse and *Ipt* reverse, S2 Table). The resulting PCR products were cloned using the pGEM-T Easy Vector (Promega, Fitchburg, USA) and transformed into the *E. coli* strain MRF (Agilent Technologies, Santa Clara, USA). At least three independent clones were sequenced to determine the transcription start site of each gene.

### Reverse transcription polymerase chain reaction (RT-PCR) and quantitative real-time PCR (qRT-PCR)

Total RNA from approximate 50 mg plant tissue was extracted by using the RNeasy Plant Mini Kit (Qiagen, Hilden, Germany) following the manufacturer’s protocol. Before reverse transcription, about 500 to 1000 ng of total RNA extracted from *Arabidopsis* tissue was digested by DNase I (Fermentas, Thermo, Waltham, USA) for 30 min at 37°C. DNase digestion was terminated by the addition of 25 mM EDTA and subsequent incubation at 70°C for 10 min. First strand cDNA synthesis was performed using oligo(dT) 18 primers (Fermentas, Thermo, Waltham, USA) and the Thermo Scientific RevertAid First Strand cDNA Synthesis Kit (Thermo, Waltham, USA). Quantitative RT-PCR with the plant cDNA samples was performed as described in [20]. The primer sequences used are listed in S2 Table.

### Protoplast transactivation (PTA) system and luminescence measurements

The Arabidopsis mesophyll protoplast isolation and transfection procedures were performed as described in [48,71]. For transfection, 30 μL protoplast suspension (approximately 1×10^4^ cells), 1 μg plasmid DNA of oncogene promoter-LUC constructs (*IaaH* promoter-LUC, *IaaM* promoter-LUC and *Ipt* promoter-LUC) and 1 μg of the expression plasmids containing the CaMV35S::transcription factor constructs of the transcription factor library [48] were combined in each well of a microtiter plate (Nunc U96; MicroWell Polypropylene Plates, Thermo, Waltham, USA). As an internal standard, 1 μg plasmid expressing the Renilla luciferase driven by the CaMV35S promoter (CaMV35S::Renilla LUC) was co-transfected. The protoplast suspension mixture was incubated overnight in the dark and at room temperature. The following day, a dual luciferase measurement was performed using the Renilla-Juice BIG Kit and Beetle-Juice BIG Kit (PJK GmbH, Kleinblittersdorf, Germany). The protoplasts settled at the bottom of the wells by gravity, then the supernatant was removed from the protoplast suspensions and 20 μL Lysis-Juice 2 (Renilla-Juice BIG KIT) was added to each well and mixed by pipetting. After 15 min on ice, the microtiter plate was centrifuged (4000 rpm for 10 min). An aliquot of 10 μL of the supernatant was transferred into the wells of two new microtiter plates. As substrate for the two types of luciferase enzymes, 50 μL Renilla-Juice for renilla luciferase (CaMV35S::Renilla LUC) and 50 μL Beetle-Juice for firefly luciferase (*IaaH* promoter-LUC, *IaaM* promoter-LUC and *Ipt* promoter-LUC) were added via the liquid handling robotic device and the luminescence was measured by the Robion Solaris plate reader luminometer (STRATEC Biomedical Systems AG, Birkenfeld, Germany). The relative luminescence intensity was calculated from the values of Firefly-LUC versus Renilla-LUC. The relative luminescence intensity calculated from the oncogene promoter-LUC constructs (*IaaH* promoter-LUC, *IaaM* promoter-LUC and *Ipt* promoter-LUC) in the absence of any expression plasmids containing the CaMV35S::transcription factor constructs or phytohormone treatments was set to 1. The fold induction in luminescence represents the relative activity induced by certain transcription factors or treatments.

### Protein expression and electrophoretic mobility shift assays (EMSA)

Protein synthesis was induced in the bacterial suspension of the transgenic *E. coli* SoluBL21 strain (Genlantis, San Diego, USA) expressing the 6×His-WRKY40 fusion protein by adding 0.5 mM Isopropyl β-D-1-thiogalactopyranoside (IPTG) at OD_600_ 0.6 overnight at 16°C. Purification of the histidine-tagged WRKY40 protein was performed according to the protocol from Novagen (Merck Millipore, Darmstadt, Germany). To generate the 50 bp of the *Ipt* promoter probe used in EMSA, two complementary oligonucleotides were synthesized by Sigma (Sigma Aldrich, St. Louis, USA). The two oligonucleotides were mixed at a 1:1 molar ratio in annealing buffer (10 mM Tris, pH8.0, 1 mM EDTA, 50 mM NaCl). The mixture was incubated at 95°C for 5 min and slowly cooled to room temperature and incubated overnight. The double-stranded oligonucleotides were purified from an 3% (w/v) agarose gel after electrophoresis, then radioactively labeled by using T4 polynucleotide kinase (Fermentas, Thermo, Waltham, USA) and [gamma-^32^P] adenosine 5’-triphosphate (ATP; Hartmann Analytic GmbH, Braunschweig, Germany). About 5 ng labeled probe and 150−300 ng 6×His-WRKY40 protein were mixed in DNA-protein binding buffer [10 mM Tris-HCl pH8.0, 0.5 mM ZnSO_4_, 0.25 mM DTT, 0.1 μg/μL poly [dI-dC], 5% (v/v) glycerol]. The binding reaction mixture was incubated on ice for 30 min and separated in a 6% (w/v) native polyacrylamide gel [45 mM Tris-borate, 1 mM EDTA, pH 8.6, 15% (v/v) Rotiphorese Gel 40 (29:1; Roth, Karlsruhe, Germany), 0.1% (w/v) ammonium persulfate (APS), 0.5% (v/v) TEMED] at 4°C for 3 h at 200 V in 0.5 × TBE buffer (45 mM Tris-borate and 1 mM EDTA; pH 8.6). The gel was fixed in 5% acetic acid for 10 min and dried for approximately 1 h (gel drying systems, Bio-Rad, Hercules, USA), exposed at −70°C to an x-ray film (Eastman Kodak, Rochester, USA) overnight and then developed.

### Cytokinin analysis

Crown gall materials used for cytokinin analysis were obtained from Wassilewskija (WS-2) stems inoculated with *A. tumefaciens* strain C58. The analysis was performed as described in [19].

### Bimolecular fluorescence complementation (BiFC) assay and microscopy

For the BiFC assay, 20 μg of each cYFP and nYFP protein fusion constructs (WRKY-cYFP, WRKY-nYFP, ARF-cYFP, ARF-nYFP and ARF5Δ722-cYFP) were transfected into mesophyll protoplasts using the PEG-calcium transfection method [71]. After incubation for 16–18 h in the dark at room temperature, protoplasts were inspected and images were taken using a confocal laser scanning microscope (Leica TCS SP5II, Leica Wetzlar, Germany).

Fluorescing plant cells and tissues were inspected and documented using an epifluorescence microscope (BZ 8000K, Biozero, Keyence, Osaka, Japan) and the software program (BZ observation application). For the inspection of intact plants, a dissecting microscope (Leica MZ6, Leica, Wetzlar, Germany) was used and pictures of crown galls were taken using a Leica DFC500 camera (Leica, Wetzlar, Germany).

### Accession numbers of *Arabidopsis* genes

The *Arabidopsis* gene indexes (AGI) of genes mentioned in the text are AT2G33860 (*ARF3*), AT1G19850 (*ARF5*), AT3G16857 (*ARR1*), AT1G10470 (*ARR4*), AT1G04550 (*IAA12*), AT5G63790 (*NAC102*), AT4G31800 (*WRKY18*), AT1G80840 (*WRKY40*), AT4G23810 (*WRKY53*) and AT2G25000 (*WRKY60*). AGI codes are from The *Arabidopsis* Information Resource database (TAIR, http://www.arabidopsis.org).

## Supporting Information

S1 Fig Alignment of *IaaH* promoters including the 5’ untranslated regions from different *A. tumefaciens* strains. Nucleotide sequences of indole-3-acetamide hydrolase (IaaH) promoters including the 5’ untranslated regions of T-DNAs from the nopaline-type (pTiC58, pTiSAKURA), octopine-type (pTi15955, pTiA6NC, pTiAch5) and agropine-type (pTiBo542) Ti plasmids. The arrow indicates the position of the transcription start sites (TSS) +1. Negative numbers indicate the nucleotide positions upstream and positive numbers downstream of the TSSs. TATA box and CAAT box sequences are written above the aligned sequences.
(PDF)

S2 Fig Alignment of *IaaM* promoters including the 5’ untranslated regions from different *A. tumefaciens* strains. Nucleotide sequences of tryptophan monooxygenase (IaaM) promoters including the 5’ untranslated regions of T-DNAs from the T-DNAs of the nopaline-type (pTiC58, pTiSAKURA), octopine-type (pTi15955, pTiA6NC, pTiAch5) and agropine-type (pTiBo542) Ti plasmids. An arrow indicates the position of the transcription start sites (TSS) +1. Negative numbers indicate the nucleotide positions upstream and positive numbers downstream of the TSSs. TATA box and CAAT box sequences are written above the aligned sequences.
(PDF)

S3 Fig Alignment of *Ipt* promoters including the 5’ untranslated regions (5’ UTR) from different *A. tumefaciens* strains. Nucleotide sequences of isopentenyl transferase (Ipt) promoters including the 5’ untranslated regions of T-DNAs from the nopaline-type (pTiC58, pTiT37, pTiSAKURA), octopine-type (pTi15955, pTiA6NC, pTiAch5) and agropine-type (pTiBo542) Ti plasmids. An arrow indicates the position of the transcription start sites (TSS). Negative numbers indicate the nucleotide positions upstream and positive numbers downstream of the TSSs. TATA box and CAAT box sequences are written above the aligned sequences. The framed “TATAAA” sequence indicates the TATA box, only conserved in the octopine type Ti plasmids. The sequence from −150 to −91 shows the conserved region, which is named as *Ipt* element.
(PDF)

S4 Fig The effects of WRKY18, WRKY40 and WRKY60 on *Ipt* promoter activity. Fold induction of *Ipt* promoter-driven luminescence in the presence of WRKY18, WRKY40 and WRKY60 transcription factor expression plasmids in the protoplast transactivation system. The relative luminescence induced by the *Ipt* promoter in protoplasts without transfection of any of the transcription factor expression plasmids was set to 1. Bars show mean values (±SD) of three independent experiments.
(PDF)

S5 Fig *Cis*-regulatory elements within IGR1 and IGR2. Positions of TATA boxes (TATAAA, blue bars), auxin responsive elements (AuxREs, TGTCNC or TGTCTN, green bars), W-boxes (TGAC, red bars) and transcript start site (TSS arrow) in the sense (above the line) and anti-sense strand (below the line) of the intergenic region (IGR1) and IGR2.
(PDF)

S6 Fig *Ipt, ARF5* and *IAA12 gene* expression. (A) Relative transcript numbers of the Ipt oncogene in 25-day-old crown galls of wild-type plants and wrky18, wrky40 and wrky60 single mutants, (B) in stems of wild-type plants 2 days and 6 days post-inoculation (2 dpi and 6 dpi) of A. tumefaciens strain C58 and (C) of ARF5 and IAA12 in crown gall tumors of the wild-type Col-0 and wrky single mutants. Relative transcript numbers were quantified by qRT-PCR and normalized to 10,000 molecules of ACTIN2/8. Bars show mean values (±SD) of three independent samples. NS: not significant. * *p-value* < 0.05; ** *p-value* < 0.01; *** *p-value* < 0.001; NS: not significant (Student’s t-test).
(PDF)

S7 Fig ARR1 and ARR4 do not activate the oncogene promoters. (A) Relative transcript numbers of the *ARR1* and *ARR4* genes in crown galls 25 days after inoculation with the virulent *A. tumefaciens* strain C58 (C58 Crown gall) and in stems inoculated with the disarmed strain GV3101 (GV3101 Stems). Relative transcript numbers were quantified by qRT-PCR and normalized to 10,000 molecules of *ACTIN2/8*. Bars show mean values (±SD) of three independent samples. (B) Fold induction of *IaaH, IaaM, Ipt* promoter-driven luminescence in *Arabidopsis* mesophyll protoplasts transfected with ARR1 and ARR4 transcription factor expression plasmids and in the presence or absence of *trans*-zeatin. The relative luminescence in the absence of ARR1, ARR4 expression plasmids and *trans*-zeatin was set to 1. Bars show mean values (±SD) of three independent experiments.
(PDF)

S1 Table List of transcription factor genes differentially expressed upon infection with *Agrobacterium tumefaciens* strain C58. Analyses are based on existing microarray data [20,46] using the Plant Transcription Factor Database v3.0 [47] (http://planttfdb.cbi.pku.edu.cn/index.php?sp=Ath) for annotation. Genes are listed according to the following criteria: Fold change (FCh) ≥ 2 or ≤ 0.5, p value < 0.01. hpi: hours post inoculation; dpi: days post inoculation.
(PDF)

S2 Table List of primers used in the different experiments. (PDF)
