# Supplementary material for: Regulation of Oncogene Expression in T-DNA-Transformed Host Plant Cells
Source: PLoS Pathog. 2015 Jan 23;11(1):e1004620. doi: 10.1371/journal.ppat.1004620 (PMC4304707; doi:10.1371/journal.ppat.1004620)
Supplement: S1 Fig — Nucleotide sequences of indole-3-acetamide hydrolase (IaaH) promoters including the 5’ untranslated regions of T-DNAs from the nopaline-type (pTiC58, pTiSAKURA), octopine-type (pTi15955, pTiA6NC, pTiAch5) and agropine-type (pTiBo542) Ti plasmids. The arrow indicates the position of the transcription start sites (TSS) +1. Negative numbers indicate the nucleotide positions upstream and positive numbers downstream of the TSSs. TATA box and CAAT box sequences are written above the aligned sequences. (PDF) [file ppat.1004620.s001.pdf]

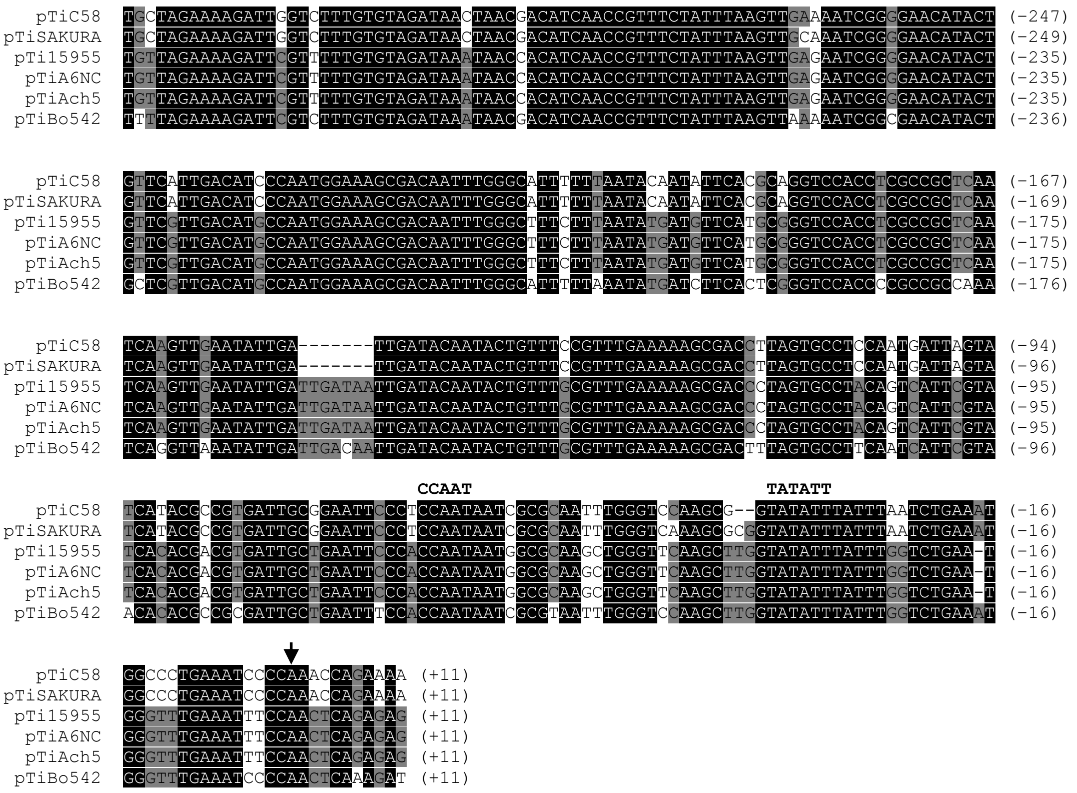

Figure S1 Alignment of *IaaH* promoters including the 5’ untranslated regions from different *A. tumefaciens* strains.

Nucleotide sequences of indole-3-acetamide hydrolase (IaaH) promoters including the 5’ untranslated regions of T-DNAs from the nopaline-type (pTiC58, pTiSAKURA), octopine-type (pTi15955, pTiA6NC, pTiAch5) and agropine-type (pTiBo542) Ti plasmids. The arrow indicates the position of the transcription start sites (TSS) +1. Negative numbers indicate the nucleotide positions upstream and positive numbers downstream of the TSSs. TATA box and CAAT box sequences are written above the aligned sequences.
