# Supplementary material for: Regulation of Oncogene Expression in T-DNA-Transformed Host Plant Cells
Source: PLoS Pathog. 2015 Jan 23;11(1):e1004620. doi: 10.1371/journal.ppat.1004620 (PMC4304707; doi:10.1371/journal.ppat.1004620)
Supplement: S3 Fig — Nucleotide sequences of isopentenyl transferase (Ipt) promoters including the 5’ untranslated regions of T-DNAs from the nopaline-type (pTiC58, pTiT37, pTiSAKURA), octopine-type (pTi15955, pTiA6NC, pTiAch5) and agropine-type (pTiBo542) Ti plasmids. An arrow indicates the position of the transcription start sites (TSS). Negative numbers indicate the nucleotide positions upstream and positive numbers downstream of the TSSs. TATA box and CAAT box sequences are written above the aligned sequences. The framed “TATAAA” sequence indicates the TATA box, only conserved in the octopine type Ti plasmids. The sequence from −150 to −91 shows the conserved region, which is named as Ipt element. (PDF) [file ppat.1004620.s003.pdf]

```
     pTiC58 TCTACGGATCCTGTTACAAGTATTGCACGTTTTATAAATTGCATATTAATGCAATCTTGATTTTTAACAACGAAGGTAAT (-575)
     pTiT37 -----GGATCCTGTTACAAGTATTGCACGTTTTATAAATTGCATATTAATGCAATCTTGATTTTCAACAACGAAGGTAAT (-535)
 pTiSAKURA TCTACGGATCCTGTTACAAGTATTGCACGTTTTATAAATTGCATATTAATGCAATCTTGATTTTCAACAACGAAGGTAAT (-575)
   pTi15955 TCTATGGATCCTGTTACAAGTATTGCAAGTTTTATAAATTGCATATTAATGCAATCTTGATTTTTAACAACGAACGTAAT (-573)
    pTiA6NC TCTATGGATCCTGTTACAAGTATTGCAAGTTTTATAAATTGCATATTAATGCAATCTTGATTTTTAACAACGAACGTAAT (-573)
    pTiAch5 TCTATGGATCCTGTTACAAGTATTGCAAGTTTTATAAATTGCATATTAATGCAATCTTGATTTTTAACAACGAACGTAAT (-573)
   pTiBo542 TCTATGGATCCCGTTACAAGTATTGCACGTTTTGTAAATTGCATATTAATGCAATCTGGATGTTTAATAACGAATGTAAT (-570)

     pTiC58 GGCGTAAAATAAAAATGTATGTTATTGTATTGATCTTTCATGATGTTGAAGTGTGCCATAATATGATGATGTATAATTA (-495)
     pTiT37 GGCGTAAAAGAAAAATGTATGTTATTGTATTGATCTTTCATGATGTTGAAGCGTGCCATAATATGATGATGTATAATTA (-455)
  pTiSAKURA GGCGTAAAAGAAAAATGTATGTTATTGTATTGATCTTTCATGATGTTGAAGCGTGCCATAATATGATGATGTATAATTA (-495)
   pTi15955 GGCGTAAA-----AAATGTATGTTATATTATTTATATTTAATTATATTGGAGTGCGCCATAATATGATGATTTATAATTA (-498)
    pTiA6NC GGCGTAAA-----AAATGTATGTTATATTATTTATATTTAATTATATTGGAGTGCGCCATAATATGATGATTTATAATTA (-498)
    pTiAch5 GGCGTAAA-----AAATGTATGTTATATTATTTATATTTAATTATATTGGAGTGCGCCATAATATGATGATTTATAATTA (-498)
   pTiBo542 GGCGTAGA----AATATGTATTTTATTGTATTTATCTTTCACTATGTTGAAGTTTGCAATAATATGCTAATGTAAAATTA (-494)

     pTiC58 AAATATTA---ACTGTCGCATTT-ATTGAAATGGCACTGTTATTTCAACCAT---ATCTT--TGATTCTGTTACAATGAC (-424)
     pTiT37 AAATATTA---ACTGTCGCATTTTATTGAAATGGCACTGTTATTTCAACCAT---ATCTT--TGATTCTGTTACA-TGAC (-384)
  pTiSAKURA AAATATTA---ACTGTCGCATTT-ATTGAAATGGCACTGTTATTTCAACCAT---ATCTT--TGATTCTGTTACAATGAC (-424)
   pTi15955 AAAAAT-ATTTACTGTCACATTG-ACTGAGATGGCACTGTTATTTCAACCATGAAATTTTGTTGATTTTTTTACAATAAC (-420)
    pTiA6NC AAAAAT-ATTTACTGTCACATTG-ACTGAGATGGCACTGTTATTTCAACCATGAAATTTTGTTGATTTTTTTACAATAAC (-420)
    pTiAch5 AAAAAT-ATTTACTGTCACATTG-ACTGAGATGGCACTGTTATTTCAACCATGAAATTTTGTTGATTTTTTTACAATAAC (-420)
   pTiBo542 AAAAATTATGTACTGCCGCATTT-GTTCAAATGGCGCCGTTATTTCAAAAAT---ATCTT--TGATTTTGTTACGATGAC (-420)

     pTiC58 AACGACTGAAAAAAGTAAATAATAGACGCCGTTGTTAAAGAATTGCTATCATATGTGCCAAACTAGAGGGAAATTTACGT (-344)
     pTiT37 A-CGACTGCAAGAAGTAAATAATAGACGCCGTTGTTAAAGAATTGCTATCATATGTGCCTAACTAGAGGGAAT------- (-312)
  pTiSAKURA AACGACTGCAAGAAGTAAATAATAGACGCCGTTGTTAAAGAATTGCTATCATATGTGCCTAACTAGAGGGAAATTTACGT (-344)
   pTi15955 AATAATTGCAGGAAGTAAATAATAGACGCCGTTGTTAAAAAATTGCAATCATATGTGCCTAACTATAGGGACAATTAAGT (-340)
    pTiA6NC AATAATTGCAGGAAGTAAATAATAGACGCCGTTGTTAAAAAATTGCAATCATATGTGCCTAACTATAGGGACAATTAAGT (-340)
    pTiAch5 AATAATTGCAGGAAGTAAATAATAGACGCCGTTGTTAAAAAATTGCAATCATATGTGCCTAACTATAGGGACAATTAAGT (-340)
   pTiBo542 AACGACTGCAGGAAGTAAATAAAAGACGCTGTTGTTAAGAAATTGCTATCATATGTGCCCAGCTATAGGGCCATTTAAGT (-344)

     pTiC58 CAATTGTGAAATAGTCGCCCTTATTTTGACGTCTCACCTAATCAAATATTACAAAAGATCTCACTCTGTCGCCAGCAATG (-264)
     pTiT37 ---TTG------AG---------------CGTCAGACCTAATCAAATATTACAAAATATCTCACTCTGTCGCCAGCAATG (-263)
  pTiSAKURA CAATTGTGAAATAGTCGCCCTTATTTTGACGTCTCACCTAATCAAATATTACAAAATATCTCACTCTGTCGCCAGCAATG (-264)
   pTi15955 CAATTGT--AATAGTCTCCCTTATTTTAACGACTCACCTAATCAAGTATTACAAAATATCTCACTTT-TCGTCAGTAATG (-263)
    pTiA6NC CAATTGT--AATAGTCTCCCTTATTTTAACGACTCACCTAATCAAGTATTACAAAATATCTCACTTT-TCGTCAGTAATG (-263)
    pTiAch5 CAATTGT--AATAGTCTCCCTTATTTTAACGACTCACCTAATCAAGTATTACAAAATATCTCACTTT-TCGTCAGTAATG (-263)
   pTiBo542 CAATTGTGAAATAGCCGCCCTTATTTTGACGTCTCATCAAATCAAATATTAAAAAATATCTCACTCTGTCGCCAGCAATG (-264)

     pTiC58 GTGTAATCAGCGCAGACAAGTGGCAGTAAAG--TGCGGAAAAACGTCCCCGAGTGGCATGAATAGCTGCCTCTGTATTGC (-186)
     pTiT37 GTGTAATCAGCGCAGACAAATGGC-GTAAAGATCGCGGAAAAACCTCCCCGAGTGGCATGA-TAGCTGCCTCTGTATTGC (-185)
  pTiSAKURA GTGTAATCAGCGCAGACAAATGGCAGTAAAG--CGCGGAAAAACGTCCCCGAGTGGCATGAATAGCTGCCTCTGTATTGC (-186)
   pTi15955 ATGTAATCAGAACTGAATAGTACAAGTAAAA--CGTGGAAAAACGTCATAGAGTGGCATGATTATATTCCTCTGCATTGC (-185)
    pTiA6NC ATGTAATCAGAACTGAATAGTACAAGTAAAA--CGTGGAAAAACGTCATAGAGTGGCATGATTATATTCCTCTGCATTGC (-185)
    pTiAch5 ATGTAATCAGAACTGAATAGTACAAGTAAAA--CGTGGAAAAACGTCATAGAGTGGCATGATTATATTCCTCTGCATTGC (-185)
   pTiBo542 ATGTAATAACCGCAGAAAAGTGAGAGTAAAT--CGCGGAAAAACGTCGCCGAGTGGCATGAATAGCGGCCTCCGTATTGC (-186)

                                               TCGTTAGTGACAAATTGCTTTCAAGGAGACAGCCATGCCCCACAC
     pTiC58 TGATTTAGTCAGCCTTATTTGACTTAAGGGTGCCCTCGTTAGTGACAAATTGCTTTCAAGGAGACAGCCATGCCCCACAC (-106)
     pTiT37 TGATTTAGTCAGCCTTATTTGACTTAAGGGTGCCCTCGTTAGTGACAAATTGCTTTCAAGGAGACAGCCATGCCCCACAC (-105)
  pTiSAKURA TGATTTAGTCAGCCTTATTTGACTTAAGGGTGCCCTCGTTAGTGACAAATTGCTTTCAAGGAGACAGCCATGCCCCACAC (-106)
   pTi15955 CAATTTATTCAGCTTTATTTGACTTAGGTGTGCCTTCGTTAGTGACAAATTGCTTTCAAGGAGACAGCCATGCCCCACAC (-105)
    pTiA6NC CAATTTATTCAGCTTTATTTGACTTAGGTGTGCCTTCGTTAGTGACAAATTGCTTTCAAGGAGACAGCCATGCCCCACAC (-105)
    pTiAch5 CAATTTATTCAGCTTTATTTGACTTAGGTGTGCCTTCGTTAGTGACAAATTGCTTTCAAGGAGACAGCCATGCCCCACAC (-105)
   pTiBo542 TGATTTAGTCAGCTTTATTTGACTTAAGGGTGCCCTCGTTAGTGACAAATTGCTTTCAAGGAGACAGCCATGCCCCACAC (-106)

            TTTGTTGAAAAACAA                  GGTAAAGCC              AAGGAATCT            TAT
     pTiC58 TTTGTTGAAAAACAAATTGCCCTTTGGGGAGACGGTAAAGCCAGTTGCTCTTCAATAAGGAATCTCGAGGAGGC-AATAT  (-27)
     pTiT37 TTTGTTGAAAAACAAATTGCC-TTTGGGGAGACGGTAAAGCCAGTTGCTCTTCAATAAGGAATCTCGAGGAGGC-AATAT  (-27)
  pTiSAKURA TTTGTTGAAAAACAAATTGCCCTTTGGGGAGACGGTAAAGCCAGTTGCTCTTCAATAAGGAATCTCGAGGAGGC-AATAT  (-27)
   pTi15955 TTTGTTGAAAAACAAGTTGCCTTTTGGG-ATACGGTAAAGCCAGTTGCACTTCAATAATGAATTTCAAGGAGAC-AATAT  (-27)
    pTiA6NC TTTGTTGAAAAACAAGTTGCCTTTTGGG-ATACGGTAAAGCCAGTTGCACTTCAATAATGAATTTCAAGGAGAC-AATAT  (-27)
    pTiAch5 TTTGTTGAAAAACAAGTTGCCTTTTGGG-ATACGGTAAAGCCAGTTGCACTTCAATAATGAATTTCAAGGAGAC-AATAT  (-27)
   pTiBo542 TTTGTTGAAAAACAAGTTGCCTTTTGGGAAGAACCTAAAGCCACTTGCTCTTCAAGGAGGAATATCGAGGAAGAGAATAT  (-26)

            AAC                        ↓ TATAAA
     pTiC58 AACCGCCTCTGGTAGTACACTTCTCTAATCCAAAAAG-TCAATTTGTATTCAAGATACCGCAAAAAACTT (+43)
     pTiT37 AACCGCCTCTGGTAGTACACTTCTCTAATCCAAAAA--TCAATTTGTATTCAAGATACCGCAAAAAACTT (+42)
  pTiSAKURA AACCGCCTCTGGTAGTACACTTCTCTAATCCAAAAA--TCAATTTGTATTCAAGATACCGCAAAAAACTT (+42)
   pTi15955 AACCGCCTCTGATAACACAATTCTCTAATATAAAAA--TCAGTTTGTATTCAATATACTGCAAAAAACTT (+42)
    pTiA6NC AACCGCCTCTGATAACACAATTCTCTAATATAAAAA--TCAGTTTGTATTCAATATACTGCAAAAAACTT (+42)
    pTiAch5 AACCGCCTCTGATAACACAATTCTCTAATATAAAAA--TCAGTTTGTATTCAATATACTGCAAAAAACTT (+42)
   pTiBo542 AACAGCCTCTGGTAC-AGACTTCTCTTGTGCAAAAAAATCAATTTGTATTCAACATATCGCAAGACCGAT (+44)
```

Figure S3 Alignment of *Ipt* promoters including the 5' untranslated regions (5' UTR) from different *A. tumefaciens* strains.

Nucleotide sequences of isopentenyl transferase (Ipt) promoters including the 5' untranslated regions of T-DNAs from the nopaline-type (pTiC58, pTiT37, pTiSAKURA), octopine-type (pTi15955, pTiA6NC, pTiAch5) and agropine-type (pTiBo542) Ti plasmids. An arrow indicates the position of the transcription start sites (TSS). Negative numbers indicate the nucleotide positions upstream and positive numbers downstream of the TSSs. TATA box and CAAT box sequences are written above the aligned sequences. The framed "TATAAA" sequence indicates the TATA box, only conserved in the octopine type Ti plasmids. The sequence from −150 to −91 shows the conserved region, which is named as *Ipt* element.
