# Supplementary material for: Regulation of Oncogene Expression in T-DNA-Transformed Host Plant Cells
Source: PLoS Pathog. 2015 Jan 23;11(1):e1004620. doi: 10.1371/journal.ppat.1004620 (PMC4304707; doi:10.1371/journal.ppat.1004620)
Supplement: S4 Fig — Fold induction of Ipt promoter-driven luminescence in the presence of WRKY18, WRKY40 and WRKY60 transcription factor expression plasmids in the protoplast transactivation system. The relative luminescence induced by the Ipt promoter in protoplasts without transfection of any of the transcription factor expression plasmids was set to 1. Bars show mean values (±SD) of three independent experiments. (PDF) [file ppat.1004620.s004.pdf]

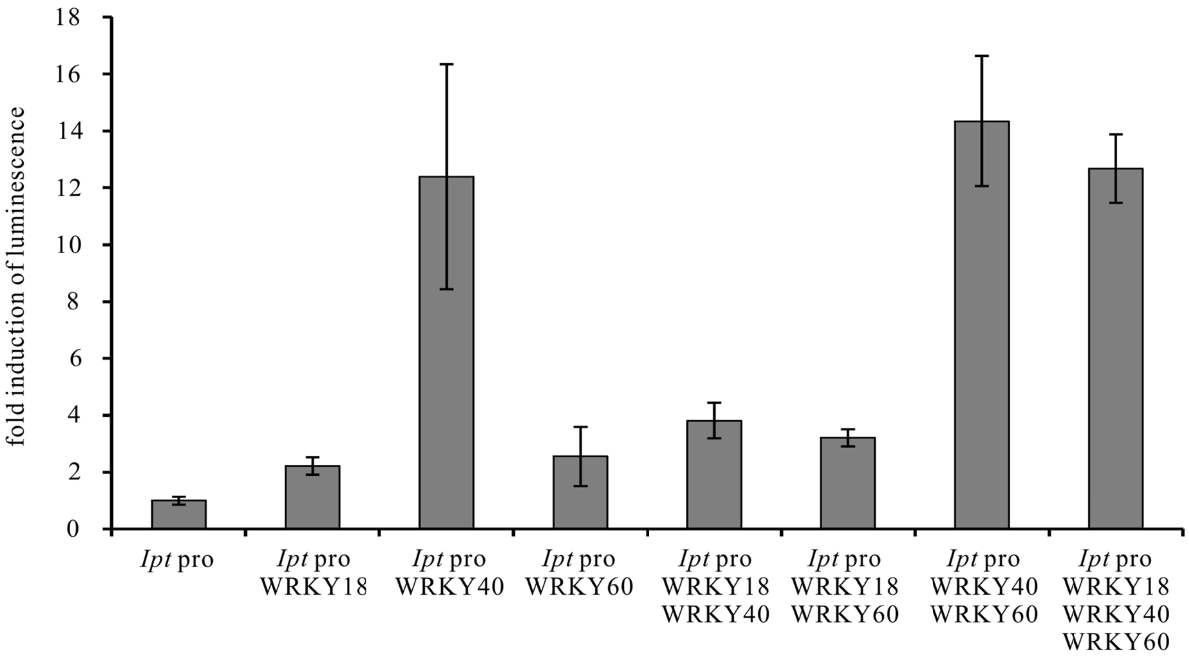

Figure S4 The effects of WRKY18, WRKY40 and WRKY60 on *Ipt* promoter activity.

Fold induction of *Ipt* promoter-driven luminescence in the presence of WRKY18, WRKY40 and WRKY60 transcription factor expression plasmids in the protoplast transactivation system. The relative luminescence induced by the *Ipt* promoter in protoplasts without transfection of any of the transcription factor expression plasmids was set to 1. Bars show mean values (±SD) of three independent experiments.
