# Supplementary material for: Regulation of Oncogene Expression in T-DNA-Transformed Host Plant Cells
Source: PLoS Pathog. 2015 Jan 23;11(1):e1004620. doi: 10.1371/journal.ppat.1004620 (PMC4304707; doi:10.1371/journal.ppat.1004620)
Supplement: S5 Fig — Positions of TATA boxes (TATAAA, blue bars), auxin responsive elements (AuxREs, TGTCNC or TGTCTN, green bars), W-boxes (TGAC, red bars) and transcript start site (TSS arrow) in the sense (above the line) and anti-sense strand (below the line) of the intergenic region (IGR1) and IGR2. (PDF) [file ppat.1004620.s005.pdf]

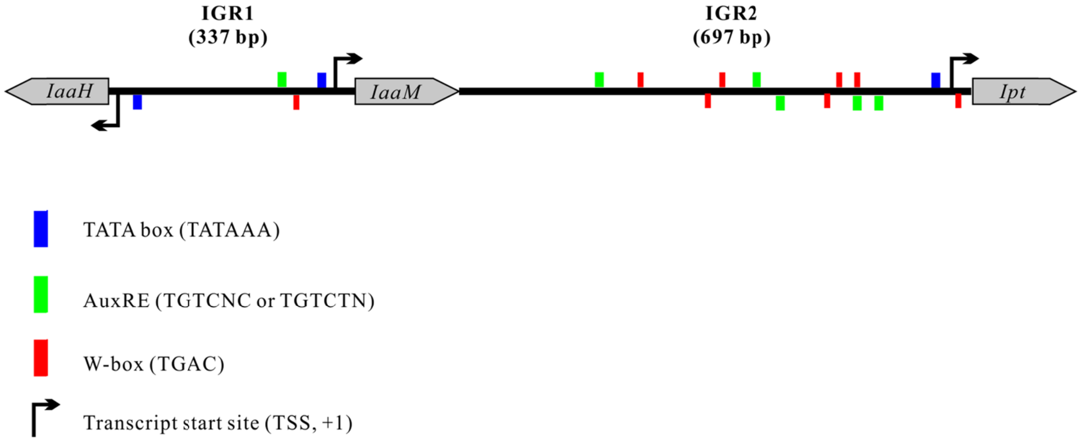

Figure S5 *Cis*-regulatory elements within IGR1 and IGR2.

Positions of TATA boxes (TATAAA, blue bars), auxin responsive elements (AuxREs, TGTCNC or TGTCTN, green bars), W-boxes (TGAC, red bars) and transcript start site (TSS arrow) in the sense (above the line) and anti-sense strand (below the line) of the intergenic region (IGR1) and IGR2.
