# Supplementary material for: Regulation of Oncogene Expression in T-DNA-Transformed Host Plant Cells
Source: PLoS Pathog. 2015 Jan 23;11(1):e1004620. doi: 10.1371/journal.ppat.1004620 (PMC4304707; doi:10.1371/journal.ppat.1004620)
Supplement: S6 Fig — (A) Relative transcript numbers of the Ipt oncogene in 25-day-old crown galls of wild-type plants and wrky18, wrky40 and wrky60 single mutants, (B) in stems of wild-type plants 2 days and 6 days post-inoculation (2 dpi and 6 dpi) of A. tumefaciens strain C58 and (C) of ARF5 and IAA12 in crown gall tumors of the wild-type Col-0 and wrky single mutants. Relative transcript numbers were quantified by qRT-PCR and normalized to 10,000 molecules of ACTIN2/8. Bars show mean values (±SD) of three independent samples. NS: not significant. * p-value < 0.05; ** p-value < 0.01; *** p-value < 0.001; NS: not significant (Student’s t-test). (PDF) [file ppat.1004620.s006.pdf]

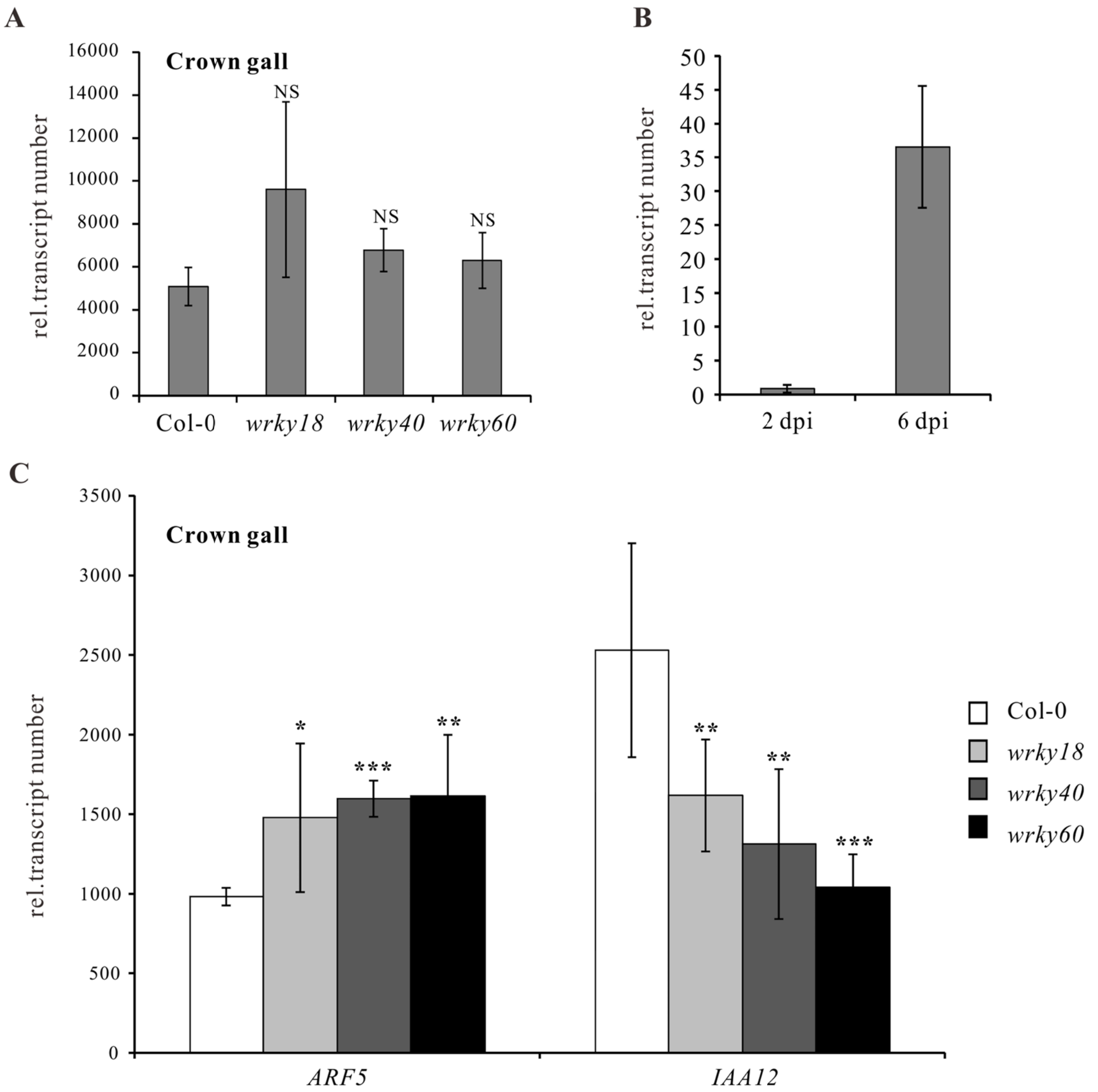

Figure S6 *Ipt, ARF5* and *IAA12* gene expression.

(A) Relative transcript numbers of the *Ipt* oncogene in 25-day-old crown galls of wild-type plants and *wrky18*, *wrky40* and *wrky60* single mutants, (B) in stems of wild-type plants 2 days and 6 days post-inoculation (2 dpi and 6 dpi) of *A. tumefaciens* strain C58 and (C) of *ARF5* and *IAA12* in crown gall tumors of the wild-type Col-0 and *wrky* single mutants. Relative transcript numbers were quantified by qRT-PCR and normalized to 10,000 molecules of *ACTIN2/8*. Bars show mean values (±SD) of three independent samples. NS: not significant. * *p-value < 0.05*; ** *p-value < 0.01*; *** *p-value < 0.001;* NS: not significant (Student's t-test).
