# Supplementary material for: Regulation of Oncogene Expression in T-DNA-Transformed Host Plant Cells
Source: PLoS Pathog. 2015 Jan 23;11(1):e1004620. doi: 10.1371/journal.ppat.1004620 (PMC4304707; doi:10.1371/journal.ppat.1004620)
Supplement: S7 Fig — (A) Relative transcript numbers of the ARR1 and ARR4 genes in crown galls 25 days after inoculation with the virulent A. tumefaciens strain C58 (C58 Crown gall) and in stems inoculated with the disarmed strain GV3101 (GV3101 Stems). Relative transcript numbers were quantified by qRT-PCR and normalized to 10,000 molecules of ACTIN2/8. Bars show mean values (±SD) of three independent samples. (B) Fold induction of IaaH, IaaM, Ipt promoter-driven luminescence in Arabidopsis mesophyll protoplasts transfected with ARR1 and ARR4 transcription factor expression plasmids and in the presence or absence of trans-zeatin. The relative luminescence in the absence of ARR1, ARR4 expression plasmids and trans-zeatin was set to 1. Bars show mean values (±SD) of three independent experiments. (PDF) [file ppat.1004620.s007.pdf]

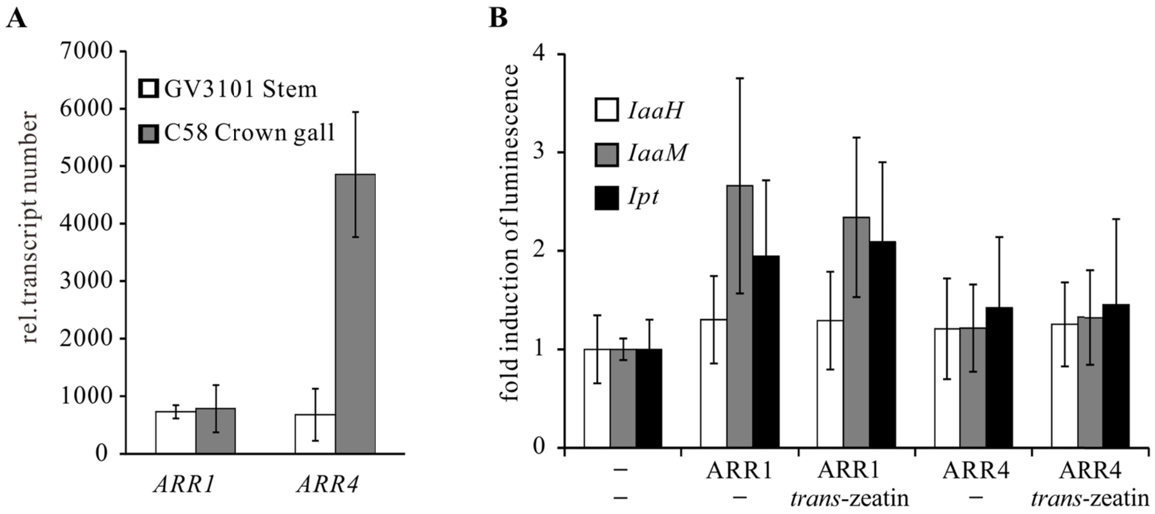

Figure S7 ARR1 and ARR4 do not activate the oncogene promoters.

(A) Relative transcript numbers of the *ARR1* and *ARR4* genes in crown galls 25 days after inoculation with the virulent *A. tumefaciens* strain C58 (C58 Crown gall) and in stems inoculated with the disarmed strain GV3101 (GV3101 Stems). Relative transcript numbers were quantified by qRT-PCR and normalized to 10,000 molecules of *ACTIN2/8*. Bars show mean values (±SD) of three independent samples. (B) Fold induction of *IaaH*, *IaaM*, *Ipt* promoter-driven luminescence in *Arabidopsis* mesophyll protoplasts transfected with ARR1 and ARR4 transcription factor expression plasmids and in the presence or absence of *trans*-zeatin. The relative luminescence in the absence of ARR1, ARR4 expression plasmids and *trans*-zeatin was set to 1. Bars show mean values (±SD) of three independent experiments.
