# Supplementary material for: Regulation of Oncogene Expression in T-DNA-Transformed Host Plant Cells
Source: PLoS Pathog. 2015 Jan 23;11(1):e1004620. doi: 10.1371/journal.ppat.1004620 (PMC4304707; doi:10.1371/journal.ppat.1004620)
Supplement: S1 Table — Analyses are based on existing microarray data [20,46] using the Plant Transcription Factor Database v3.0 [47] (http://planttfdb.cbi.pku.edu.cn/index.php?sp=Ath) for annotation. Genes are listed according to the following criteria: Fold change (FCh) ≥ 2 or ≤ 0.5, p value < 0.01. hpi: hours post inoculation; dpi: days post inoculation. (PDF) [file ppat.1004620.s008.pdf]

Supporting Tables

Table S1 List of transcription factor genes differentially expressed upon infection with *Agrobacterium tumefaciens* strain C58.

Analyses are based on existing microarray data [1,2] using the Plant Transcription Factor Database v3.0 [3] (http://planttfdb.cbi.pku.edu.cn/index.php?sp=Ath) for annotation. Genes are listed according to the following criteria: Fold change (FCh) ≥ 2 or ≤ 0.5, p value < 0.01. hpi: hours post inoculation; dpi: days post inoculation.

Locus	FCh	P Value	Family	Description
**3 hpi**				
AT4G23810	2.47	0.000	WRKY	WRKY53 (WRKY DNA-binding protein 53)
AT1G80840	2.22	0.001	WRKY	WRKY40 (WRKY DNA-binding protein 40)
AT5G63790	2.18	0.001	NAC	ANAC102 (Arabidopsis NAC domain containing protein 102)
**6 dpi**				
AT5G12330	2.76	0.000	SRS	LRP1 (LATERAL ROOT PRIMORDIUM 1)
AT5G65640	2.36	0.000	bHLH	BHLH093 (BETA HLH PROTEIN 93)
AT4G17980	2.24	0.000	NAC	ANAC071 (Arabidopsis NAC domain containing protein 71)
AT5G25190	2.23	0.000	ERF	ethylene-responsive element-binding protein, putative
AT2G47260	2.22	0.000	WRKY	WRKY23 (WRKY DNA-binding protein 23)
AT1G10585	2.15	0.000	bHLH	transcription factor
AT5G57660	0.49	0.000	CO-like	zinc finger (B-box type) family protein
AT5G61600	0.48	0.000	ERF	ethylene-responsive element-binding family protein
AT1G74840	0.48	0.000	MYB related	myb family transcription factor
AT2G28200	0.47	0.000	C_2H_2	nucleic acid binding / transcription factor/ zinc ion binding
AT5G49450	0.46	0.000	bZIP	ATBZIP1 (ARABIDOPSIS THALIANA BASIC LEUCINE-ZIPPER 1)
AT5G61590	0.35	0.000	ERF	AP2 domain-containing transcription factor family protein
**35 dpi crown gall**				
AT2G47260	11.63	0.000	WRKY	WRKY23 (WRKY DNA-binding protein 23)
AT1G31320	9.85	0.000	LBD	LBD4 (LOB DOMAIN-CONTAINING PROTEIN 4)
AT3G02550	9.63	0.000	LBD	LBD41 (LOB DOMAIN-CONTAINING PROTEIN 41)
AT5G60200	9.30	0.000	Dof	Dof-type zinc finger domain-containing protein

AT5G25190	8.72	0.000	ERF	ethylene-responsive element-binding protein, putative
AT5G12330	7.83	0.000	SRS	LRP1 (LATERAL ROOT PRIMORDIUM 1)
AT2G31180	5.64	0.000	MYB	AtMYB14/Myb14at (myb domain protein 14)
AT1G62300	5.52	0.001	WRKY	WRKY6 (WRKY DNA-binding protein 6)
AT1G01010	5.06	0.000	NAC	ANAC001 (Arabidopsis NAC domain containing protein 1)
AT1G19850	4.93	0.000	ARF	MP (MONOPTEROS)
AT3G10040	4.81	0.000	Trihelix	transcription factor
AT5G25160	4.80	0.000	C_2H_2	ZFP3 (ZINC FINGER PROTEIN 3)
AT1G05710	4.61	0.000	bHLH	ethylene-responsive protein, putative
AT3G25730	4.26	0.000	RAV	AP2 domain-containing transcription factor, putative
AT2G34710	4.15	0.000	HD-ZIP	PHB (PHABULOSA)
AT5G13330	4.10	0.000	ERF	RAP2.6L (related to AP2 6L)
AT5G47370	4.09	0.007	HD-ZIP	HAT2
AT1G68320	4.06	0.000	MYB	MYB62 (myb domain protein 62)
AT1G36060	4.05	0.000	ERF	AP2 domain-containing transcription factor, putative
AT1G06180	4.00	0.000	MYB	ATMYB13 (myb domain protein 13)
AT5G13080	3.79	0.002	WRKY	WRKY75 (WRKY DNA-BINDING PROTEIN 75)
AT2G36080	3.71	0.001	B3	DNA-binding protein, putative
AT2G01430	3.53	0.007	HD-ZIP	homeobox-leucine zipper protein 17 (HB-17) / HD-ZIP transcription factor 17
AT3G04670	3.51	0.001	WRKY	WRKY39 (WRKY DNA-binding protein 39)
AT5G61890	3.51	0.000	ERF	AP2 domain-containing transcription factor family protein
AT5G10510	3.39	0.000	AP2	AIL6 (AINTEGUMENTA-LIKE 6)
AT3G03660	3.32	0.009	WOX	DNA binding / transcription factor
AT3G01970	3.25	0.001	WRKY	WRKY45 (WRKY DNA-binding protein 45)
AT3G23240	3.23	0.004	ERF	ATERF1/ERF1 (ETHYLENE RESPONSE FACTOR 1)
AT3G61850	3.11	0.001	Dof	DAG1 (DOF AFFECTING GERMINATION 1)
AT4G37750	3.10	0.001	AP2	ANT (AINTEGUMENTA)
AT1G29160	3.07	0.005	Dof	Dof-type zinc finger domain-containing protein
AT5G03680	3.05	0.001	Trihelix	PTL (PETAL LOSS)
AT5G22570	2.97	0.006	WRKY	WRKY38 (WRKY DNA-binding protein 38)
AT5G66700	2.94	0.000	HD-ZIP	HB53 (homeobox-8)
AT1G57560	2.90	0.000	MYB	AtMYB50 (myb domain protein 50)
AT5G65510	2.88	0.000	AP2	AIL7 (AINTEGUMENTA-LIKE 7)
AT3G24120	2.88	0.000	G2-like	myb family transcription factor
AT4G32880	2.85	0.002	HD-ZIP	ATHB-8 (HOMEOBOX GENE 8)
AT2G47520	2.82	0.000	ERF	AP2 domain-containing transcription factor, putative
AT5G39610	2.76	0.004	NAC	ANAC092/ATNAC2/ATNAC6 (Arabidopsis NAC domain containing protein 92)
AT2G18550	2.71	0.008	HD-ZIP	ATHB21/HB-2 (homeobox-2)
AT5G49620	2.71	0.007	MYB	AtMYB78 (myb domain protein 78)

AT1G80730	2.68	0.003	C_2H_2	ZFP1 (ARABIDOPSIS THALIANA ZINC-FINGER PROTEIN 1)
AT5G17490	2.57	0.000	GRAS	RGL3 (RGA-LIKE 3)
AT2G41690	2.52	0.001	HSF	AT-HSFB3 (Arabidopsis thaliana heat shock transcription factor B3)
AT5G57390	2.52	0.000	AP2	AIL5 (AINTEGUMENTA-LIKE 5)
AT5G49450	2.50	0.005	GRAS	ATBZIP1 (ARABIDOPSIS THALIANA BASIC LEUCINE-ZIPPER 1)
AT5G17300	2.47	0.002	MYB related	myb family transcription factor
AT1G02220	2.46	0.004	NAC	ANAC003 (Arabidopsis NAC domain containing protein 3)
AT4G29190	2.45	0.001	C_3H	zinc finger (CCCH-type) family protein
AT2G23320	2.44	0.000	WRKY	WRKY15 (WRKY DNA-binding protein 15)
AT4G37540	2.42	0.007	LBD	LBD39 (LOB DOMAIN-CONTAINING PROTEIN 39)
AT5G15160	2.40	0.004	bHLH	bHLH family protein
AT3G45610	2.38	0.000	Dof	Dof-type zinc finger domain-containing protein
AT5G03790	2.34	0.000	HD-ZIP	ATHB51/LMI1 (LATE MERISTEM IDENTITY1)
AT1G46264	2.31	0.002	HSF	AT-HSFB4 (Arabidopsis thaliana heat shock transcription factor B4)
AT2G43000	2.28	0.001	NAC	ANAC042 (Arabidopsis NAC domain containing protein 42)
AT2G34140	2.22	0.001	Dof	Dof-type zinc finger domain-containing protein
AT1G80590	2.18	0.000	WRKY	WRKY66 (WRKY DNA-binding protein 66)
AT2G46590	2.18	0.003	Dof	DAG2 (DOF AFFECTING GERMINATION 2)
AT4G37650	2.16	0.001	GRAS	SHR (SHORT ROOT)
AT1G79180	2.15	0.000	MYB	AtMYB63 (myb domain protein 63)
AT4G17980	2.15	0.002	NAC	ANAC071 (Arabidopsis NAC domain containing protein 71)
AT1G69780	2.14	0.000	HD-ZIP	ATHB13
AT5G46590	2.12	0.002	NAC	ANAC096 (Arabidopsis NAC domain containing protein 96)
AT4G36160	2.08	0.000	NAC	ANAC076/VND2 (VASCULAR-RELATED NAC-DOMAIN 2)
AT5G39660	2.06	0.008	Dof	CDF2 (CYCLING DOF FACTOR 2)
AT2G37590	2.05	0.007	Dof	Dof-type zinc finger domain-containing protein
AT5G13910	2.05	0.006	ERF	LEP (LEAFY PETIOLE)
AT1G68840	2.05	0.001	RAV	RAV2 (REGULATOR OF THE ATPASE OF THE VACUOLAR MEMBRANE)
AT5G65790	2.05	0.001	MYB	MYB68 (myb domain protein 68)
AT1G30490	2.03	0.001	HD-ZIP	PHV (PHAVOLUTA)
AT1G69690	0.50	0.006	TCP	TCP family transcription factor, putative
AT5G07580	0.50	0.007	ERF	DNA binding / transcription factor

AT5G15850	0.50	0.006	CO-like	COL1 (CONSTANS-LIKE 1)
AT1G76890	0.50	0.003	Trihelix	GT2
AT2G35550	0.49	0.000	BBR-BPC	ATBPC7/BBR/BPC7/BPC7 (BASIC PENTACYSTEINE 7)
AT4G17460	0.49	0.001	HD-ZIP	HAT1 (homeobox-leucine zipper protein 1)
AT2G14210	0.49	0.008	MIKC	ANR1
AT3G14020	0.48	0.000	NF-YA	CCAAT-binding transcription factor (CBF-B/NF-YA) family protein
AT3G28920	0.48	0.000	ZF-HD	ATHB34 (ARABIDOPSIS THALIANA HOMEOBOX PROTEIN 34)
AT3G59580	0.48	0.000	Nin-like	RWP-RK domain-containing protein
AT1G27360	0.47	0.000	SBP	squamosa promoter-binding protein-like 11 (SPL11)
AT3G11020	0.47	0.000	ERF	DREB2B (DRE-binding protein 2B)
AT3G61890	0.46	0.006	HD-ZIP	ATHB-12 (ARABIDOPSIS THALIANA HOMEOBOX PROTEIN 12)
AT4G00050	0.46	0.004	bHLH	UNE10 (unfertilized embryo sac 10)
AT1G56170	0.46	0.007	NF-YC	HAP5B (Heme activator protein (yeast) homolog 5B)
AT2G43010	0.46	0.001	bHLH	PIF4 (PHYTOCHROME INTERACTING FACTOR 4)
AT1G30500	0.45	0.001	NF-YA	CCAAT-binding transcription factor (CBF-B/NF-YA) family protein
AT5G47640	0.45	0.004	NF-YB	CCAAT-box binding transcription factor subunit B (NF-YB) (HAP3) (AHAP3) family (Hap3b)
AT4G36930	0.44	0.001	bHLH	SPT (SPATULA)
AT1G70510	0.44	0.001	TALE	KNAT2 (KNOTTED-LIKE FROM ARABIDOPSIS THALIANA 2)
AT2G33480	0.44	0.007	NAC	ANAC041 (Arabidopsis NAC domain containing protein 41)
AT5G56860	0.43	0.000	GATA	GNC (GATA, NITRATE-INDUCIBLE, CARBON METABOLISM-INVOLVED)
AT3G21270	0.43	0.003	Dof	ADOF2 (Arabidopsis dof zinc finger protein 2)
AT1G22070	0.42	0.000	bZIP	TGA3 (TGA1a-related gene 3)
AT4G17880	0.42	0.002	bHLH	basic helix-loop-helix (bHLH) family protein
AT2G27220	0.41	0.003	TALE	BLH5 (BELL1-LIKE HOMEODOMAIN 5)
AT1G54160	0.41	0.000	NF-YA	CCAAT-binding transcription factor (CBF-B/NF-YA) family protein
AT5G18240	0.41	0.001	G2-like	MYR1 (MYB-RELATED PROTEIN 1)
AT2G02070	0.41	0.000	C_2H_2	ATIDD5 (ARABIDOPSIS THALIANA INDETERMINATE(ID)-DOMAIN 5)
AT4G08150	0.40	0.004	TALE	KNAT1 (BREVIPEDICELLUS 1)
AT1G26960	0.40	0.002	HD-ZIP	ATHB23 (ARABIDOPSIS THALIANA HOMEOBOX PROTEIN 23)
AT2G01570	0.40	0.000	GRAS	RGA1 (REPRESSOR OF GA1-3 1)
AT2G20570	0.38	0.000	G2-like	GPRI1 (GOLDEN2-LIKE 1)

AT5G28300	0.35	0.000	Trihelix	trihelix DNA-binding protein, putative
AT4G00730	0.35	0.001	HD-ZIP	ANL2 (ANTHOCYANINLESS 2)
AT2G35940	0.34	0.001	TALE	BLH1 (embryo sac development arrest 29)
AT3G11090	0.34	0.000	LBD	LBD21 (LOB DOMAIN-CONTAINING PROTEIN 21)
AT1G61660	0.34	0.000	bHLH	basic helix-loop-helix (bHLH) family protein
AT1G74660	0.33	0.000	ZF-HD	MIF1 (MINI ZINC FINGER 1)
AT2G02080	0.32	0.000	C_2H_2	ATIDD4 (ARABIDOPSIS THALIANA INDETERMINATE(ID)-DOMAIN 4)
AT5G60850	0.32	0.000	Dof	OBP4 (OBF BINDING PROTEIN 4)
AT2G33810	0.32	0.000	SBP	SPL3 (SQUAMOSA PROMOTER BINDING PROTEIN-LIKE 3)
AT4G36870	0.31	0.000	TALE	BLH2 (BEL1-LIKE HOMEODOMAIN 2, SAWTOOTH 1)
AT1G71030	0.30	0.001	MYB related	ATMYBL2 (Arabidopsis myb-like 2)
AT2G47890	0.30	0.001	DBB	zinc finger (B-box type) family protein
AT5G61420	0.30	0.001	MYB	MYB28 (MYB DOMAIN PROTEIN 28)
AT1G52880	0.29	0.001	NAC	NAM (Arabidopsis NAC domain containing protein 18)
AT5G44190	0.28	0.000	G2-like	GLK2 (GOLDEN2-LIKE 2)
AT5G62000	0.27	0.006	ARF	ARF2 (AUXIN RESPONSE FACTOR 2)
AT2G38090	0.26	0.002	MYB	myb family transcription factor
AT2G03710	0.26	0.000	MIKC	SEP4 (SEPALLATA4)
AT2G44745	0.26	0.007	WRKY	WRKY family transcription factor
AT1G18710	0.25	0.005	MYB	AtMYB47 (myb domain protein 47)
AT3G59060	0.25	0.003	bHLH	PIL6 (PHYTOCHROME-INTERACTING FACTOR 5)
AT1G55110	0.24	0.000	C_2H_2	ATIDD7 (ARABIDOPSIS THALIANA INDETERMINATE(ID)-DOMAIN 7)
AT1G32640	0.24	0.004	bHLH	ATMYC2 (JASMONATE INSENSITIVE 1)
AT4G28530	0.24	0.000	NAC	ANAC074 (Arabidopsis NAC domain containing protein 74)
AT5G60910	0.22	0.001	MIKC	AGL8 (AGAMOUS-LIKE 8)
AT3G05690	0.21	0.000	NF-YA	ATHAP2B/HAP2B/UNE8 (HEME ACTIVATOR PROTEIN (YEAST) HOMOLOG 2B)
AT3G53310	0.20	0.000	B3	transcriptional factor B3 family protein
AT5G06510	0.20	0.000	NF-YA	CCAAT-binding transcription factor (CBF-B/NF-YA) family protein
AT2G17040	0.19	0.000	NAC	ANAC036 (Arabidopsis NAC domain containing protein 36)
AT1G66390	0.19	0.006	MYB	PAP2 (PRODUCTION OF ANTHOCYANIN PIGMENT 2)
AT1G56650	0.19	0.002	MYB	PAP1 (PRODUCTION OF ANTHOCYANIN PIGMENT 1)
AT5G59780	0.16	0.001	MYB	MYB59 (myb domain protein 59)
AT1G62360	0.14	0.001	TALE	STM (SHOOT MERISTEMLESS)
AT3G46130	0.13	0.000	MYB	MYB111 (myb domain protein 111)

1. Deeken R, Engelmann JC, Efetova M, Czirjak T, Muller T, et al. (2006) An integrated view of gene expression and solute profiles of Arabidopsis tumors: a genome-wide approach. Plant Cell 18: 3617-3634.

2. Lee CW, Efetova M, Engelmann JC, Kramell R, Wasternack C, et al. (2009) Agrobacterium tumefaciens promotes tumor induction by modulating pathogen defense in Arabidopsis thaliana. Plant Cell 21: 2948-2962.

3. Jin J, Zhang H, Kong L, Gao G, Luo J (2014) PlantTFDB 3.0: a portal for the functional and evolutionary study of plant transcription factors. Nucleic Acids Res 42: D1182-1187.
