# Supplementary material for: Regulation of Oncogene Expression in T-DNA-Transformed Host Plant Cells
Source: PLoS Pathog. 2015 Jan 23;11(1):e1004620. doi: 10.1371/journal.ppat.1004620 (PMC4304707; doi:10.1371/journal.ppat.1004620)
Supplement: S2 Table — (PDF) [file ppat.1004620.s009.pdf]

Table S2 List of primers used in the different experiments

**Gene name (Accession No.)**	**Primer**	**Primer sequence (5’ ➔ 3’)**	**Product size**	**Experiment**
ACTIN2/8 (ACT2, AT3G18780, ACT8, AT1G49240)	forward	GGTGATGGTGTGTCT	435 bp	qRT-PCR
	reverse	ACTGAGCACAATGTTAC		
IaaH (pTiC58, AE007871)	forward	ACCTTGATGCTGATGTGGCC	597 bp	qRT-PCR
	reverse	CCCCGATTGCTAACAGACG		
IaaM (pTiC58, AE007871)	forward	TGCCCAGCATCTAGTT	309 bp	qRT-PCR
	reverse	CAAGAGTGTTCGAGAGG		
Ipt (pTiC58, AE007871)	forward	CAGTTATTGGAGTGCG	290 bp	qRT-PCR
	reverse	TCCCATGAATCAACTTAT		
WRKY18 (AT4G31800)	forward	AAAGTTTCGACTGTCT	325 bp	qRT-PCR
	reverse	CCAACGCTAGTCTATGA		
WRKY40 (AT1G80840)	forward	TGACTAGAGACAATCCAT	413 bp	qRT-PCR
	reverse	ATACAATTTTCCGGTAAC		
WRKY60 (AT2G25000)	forward	CCAACGATAAAGCGAC	318 bp	qRT-PCR
	reverse	TTTCCTCAACTGGTTC		
ARF5 (AT1G19850)	forward	GATAAACCTACTCGG	424 bp	qRT-PCR
	reverse	GGCCACTCGTATTAGA		
ARR1 (AT3G16857)	forward	AACTCGTTTATGACGG	337 bp	qRT-PCR
	reverse	TTCGATTACCCATAGG		
ARR4 (AT1G10470)	forward	TCCTGCAAAGTTACGG	247 bp	qRT-PCR
	reverse	CCTCAAGGCATCTGTC		
IAA12 (AT1G04550)	forward	GAATTGGGATTAGGGC	446 bp	qRT-PCR
	reverse	TCTCGACAAGTAGTACC		
**Primer Name**	**Primer**	**Primer sequence (5’ ➔ 3’)**	**Product size**	**Constructs**
Luc user	forward	GGCTTAAUTAAGGATCCTTAATTAAACCTCAG CATGGAAGACGCTAAAAACATAA	1902 bp	::LUC
CAMV Term user	reverse	GGTTTAAUATCGATCTGGATTTTAGTACTGG		
35S USER	forward	GGCTTAAUTCTAGAGATCCGTCAACATGGTGG	759 bp	35S promoter :: LUC
35S USER	reverse	GGTTTAAUTCCTCTCCAAATGAAATGAACTTCC		
IaaH pro USER	forward	GGCTTAAUTGCTAGAAAAGATTGGTCTTTGTG	337 bp	*IaaH* promoter :: LUC
IaaH pro USER	reverse	GGTTTAAUTTTTCTGGTTTGGGGATTTCAG		
IaaM pro USER	forward	GGCTTAAUTTTTCTGGTTTGGGGATTTCAG	337 bp	*IaaM* promoter :: LUC
IaaM pro User	reverse	GGTTTAAUTGCTAGAAAAGATTGGTCTTTGTG		

Ipt pro USER	forward	GGCTTAAUTCTACGGATCCTGTTACAAGTATT	697 bp	*Ipt* promoter :: LUC
Ipt pro USER	reverse	GGTTTAAUAAGTTTTTTGCGGTATCTTGAATAC		
ARF5 USER fwd	forward	GGCTTAAUATGATGGCTTCATTGTCTTGT	2706 bp	ARF5-cYFP ARF5-nYFP
ARF5 USER CC	reverse	GGTTTAAUCCTGAAACAGAAGTCTTAAGATCG		
WRKY40 USER	forward	GGCTTAAUATGGATCAGTACTCATCCTCTTTG	906 bp	WRKY40-cYFP; WRKY40-nYFP
WRKY40 USER CC	reverse	GGTTTAAUCCTTTCTCGGTATGATTCTGTTGATA		
WRKY18 USER	forward	GGCTTAAUATGGACGGTTCTTCGTTTCTC	930 bp	WRKY18-nYFP
WRKY18 USER CC	reverse	GGTTTAAUCCTGTTCTAGATTGCTCCATTAACC		
WRKY60 USER	forward	GGCTTAAUATGGACTATGATCCCAACACC	813 bp	WRKY60-nYFP
WRKY60 USER CC	reverse	GGTTTAAUCCTGTTCTTGAATGCTCTATCAATCT		
ARF3 USER fwd	forward	GGCTTAAUATGGGTGGTTTAATCGATCTGAA	1824 bp	ARF3-cYFP
ARF3 USER CC	reverse	GGTTTAAUCCGAGAGCAATGTCTAGCAACATG		
ARF5 722 USER CC	reverse	GGTTTAAUCCCAAACAACCAGAAGGGTGGTTC	2166 bp	ARF5Δ722-cYFP
WRKY53 USER	forward	GGCTTAAUATGGAAGGAAGAGATATGTTAAGTTG	972 bp	WRKY53-cYFP
WRKY53 USER CC	reverse	GGTTTAAUCCATAATAAATCGACTCGTGTAAAAACGC		
WRKY40 NdeI	forward	GGGTTTCATATGGATCAGTACTCATCCTCTTTG	909 bp	6×His::WRKY40
WRKY40 XhoI	reverse	CCGCTCGAGCTATTTCTCGGTATGATTCTGTTG		
AuxRE 1m	forward	TTAAAATATTAACTGGCGCATTTATTGAAAT	–	*Ipt* promoter AuxREm
AuxRE 1m	reverse	ATTTCAATAAATGCGCCAGTTAATATTTTAA	–	
AuxRE 2m	forward	AAAGATCTCACTCTGGCGCCAGCAATGGTGT	–	*Ipt* promoter AuxREm
AuxRE 2m	reverse	ACACCATTGCTGGCGCCAGAGTGAGATCTTT	–	
AuxRE 3m	forward	GTGTAATCAGCGCAGCCAAGTGGCAGTAAAG	–	*Ipt* promoter AuxREm
AuxRE 3m	reverse	CTTTACTGCCACTTGGCTGCGCTGATTACAC	–	
AuxRE 4m	forward	GTGCCCTCGTTAGTGCCAAATTGCTTTCAAG	–	*Ipt* promoter AuxREm
AuxRE 4m	reverse	CTTGAAAGCAATTTGGCACTAACGAGGGCAC	–	
AuxRE 5m	forward	ATTGCTTTCAAGGAGCCAGCCATGCCCCAC	–	*Ipt* promoter AuxREm
AuxRE 5m	reverse	GTGGGGCATGGCTGGCTCCTTGAAAGCAAT	–	
